# Design of Glycosyltransferase Inhibitors: Targeting the Biosynthesis of Glycosaminoglycans by Phosphonate-Xyloside

**DOI:** 10.1021/acsomega.5c06840

**Published:** 2025-11-17

**Authors:** Daniel Willén, Hanna Malmquist, Pilar Blasco, Joachim Björklund, Roberto Mastio, Sophie Manner, Göran Widmalm, Emil Tykesson, Ulf Ellervik

**Affiliations:** a Department of Chemistry, Centre for Analysis and Synthesis, 5193 Lund University, P.O. Box 124, Lund SE-221 00, Sweden; b Arrhenius Laboratory, Department of Organic Chemistry, 7675 Stockholm University, Stockholm SE-106 91, Sweden; c Department of Experimental Medical Science, 5193 Lund University, P.O. Box 117, Lund SE-221 00, Sweden

## Abstract

β-1,4-Galactosyltransferase 7 (β4GalT7) is a key enzyme in the biosynthesis of glycosaminoglycans (GAG) that transfers the first galactose unit to xylose in the linker region. Searching for new inhibitors of the GAG biosynthesis, we used saturation transfer difference (STD) nuclear magnetic resonance (NMR) spectroscopy to evaluate the binding interactions between β4GalT7 and several pentosides in the presence of UDP donors. These investigations verified the glycosylation specificity of β4GalT7 and revealed that the naphthalene and the uridine moieties were significant contributors to the binding of the acceptor and the donor, respectively, while the galactose part was less important. Based on these findings, we set out to investigate conjugates of UDP and naphthoxylosides to function as transition state analogues. These compounds were synthesized using a one-pot procedure and tested as inhibitors in a β4GalT7 assay. Interestingly, one truncated analogue, a bisphosphonate-xyloside construct, showed a significant inhibition (IC_50_: 188 μM). These findings open for the design of a new class of inhibitors of the GAG biosynthesis.

## Graphical abstract

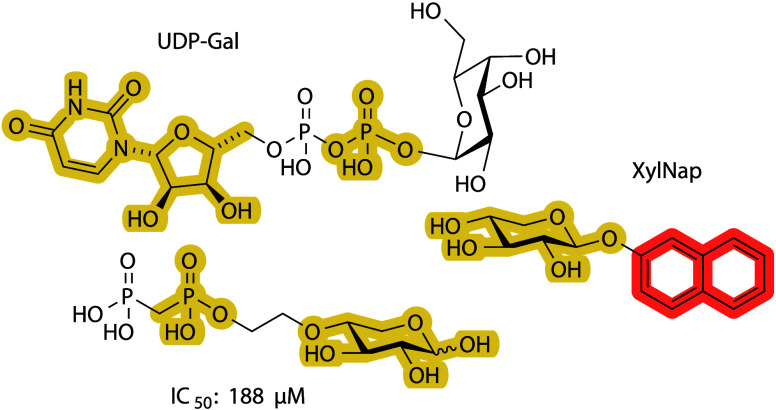

## Introduction

β-1,4-Galactosyltransferase 7 (β4GalT7), situated in the Golgi apparatus, catalyzes the transfer of galactose to a xylose residue and is thus a key step in the biosynthesis of glycosaminoglycans (GAG). GAGs are long, linear, and negatively charged polysaccharides that, connected to a core protein, form proteoglycans (PG). PGs are located mainly on the cell surface and in the extracellular matrix (ECM), and influence cell communication, growth factor regulation, inflammation, and angiogenesis.^1–3^

GAGs and PGs play important roles in cancer cell proliferation, invasion and metastasis.^4^ However, despite a growing knowledge on GAG interactions in cancer events, several processes are still not fully comprehended. For example, depending on the stage of the disease, the very same type of GAG can act as an inhibitor or a promoter of cancer growth.^5^ To shed light on the roles of GAGs in cancer, and other diseases such as infections, we are developing tools to regulate the GAG biosynthesis.

Heparan sulfate (HS) and chondroitin sulfate/dermatan sulfate (CS/DS)—the two major classes of glycosaminoglycans (GAGs)—have a common tetrasaccharide linker: GlcA­(β1–3)­Gal­(β1–3)­Gal­(β1–4)­Xylβ (Figure 1a). The initial galactosylation reaction forming the Gal­(β1–4)­Xyl linkage is catalyzed by the glycosyl transferase β4GalT7.^6^

**Figure 1:**
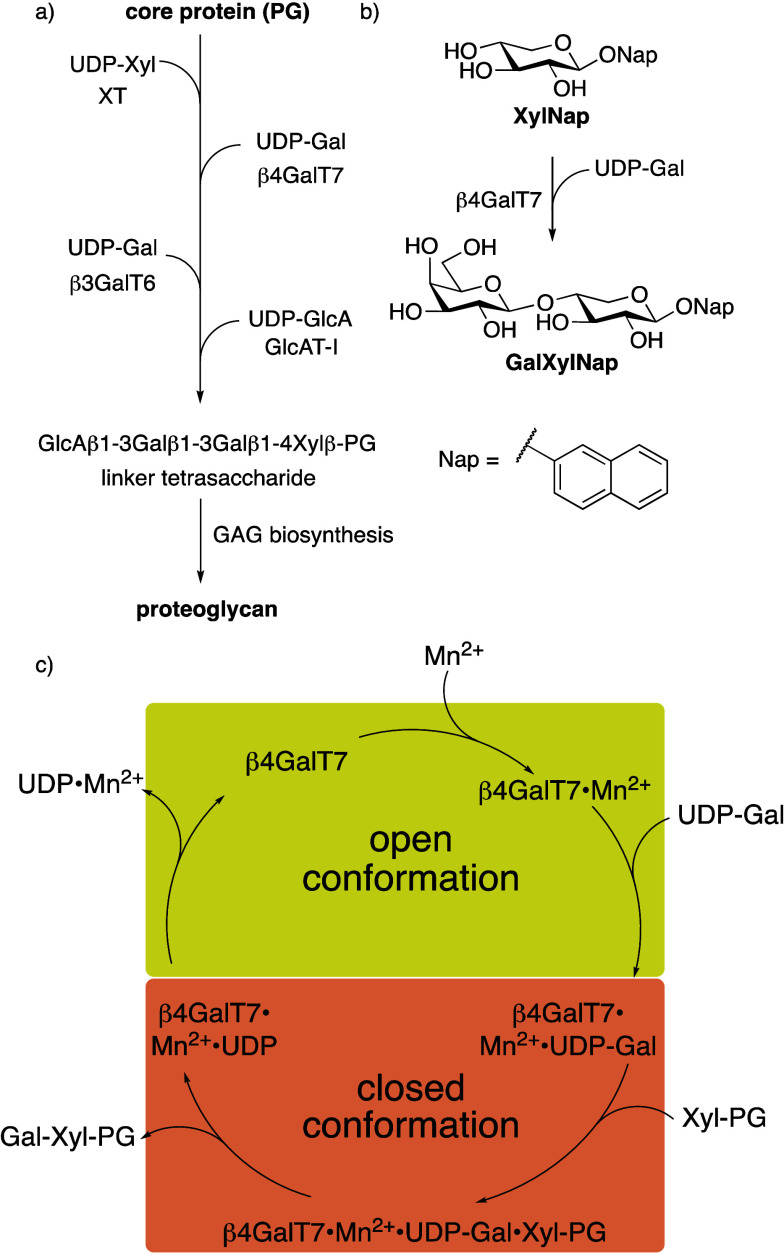
(a) Biosynthesis of the linker tetrasaccharide of HS and CS/DS. (b) Galactosylation of **XylNap** by β4GalT7 to form GalXylNap. (c) Suggested mechanistic pathway for galactosylation by β4GalT7.

Glycosyl transferases (GTs) are enzymes that transfer a sugar residue, often in the form of a UDP-sugar, to an acceptor such as a protein or another carbohydrate.^7–9^ β4GalT7 is an example of a metal ion dependent (Mn^2+^) GT. In general, GT inhibitors can be divided into several classes: nucleotide-sugar donor mimics, acceptor mimics, transition state analogs, and glycomimetics. In the transition state analogs, the donor and acceptor moieties are covalently coupled. There are few examples of such inhibitors for galactosyl transferases. In 1996 Hashimoto et al. published bisubstrate analogs with a linker between HO-2 of UDP-Gal and HO-6 of GlcNAc. These analogs showed rather strong inhibition of β-1,4-galactosyltransferases from bovine milk.^10^

One problem regarding the design of GT inhibitors is structure promiscuity of the enzyme. The same UDP-sugar donors are used by several GTs and there is a risk that the acceptor mimic may be recognized also by other GTs. For this reason, GTs are under-explored from a drug discovery perspective. From our earlier research, as well as the literature, we conclude that β4GalT7 is selective for xylosides, and that most modifications of the xylose unit result in diminished enzymatic activity. In general, methylation, fluorination or deoxygenation of position 4 of xylose, results in moderate to strong inhibitors of β4GalT7.^11–15^

Furthermore, β-d-xylopyranosides with hydrophobic aglycones can permeate cell membranes and initiate GAG synthesis in competition with the natural PG synthesis (Figure 1b). Since these GAGs are not connected to a protein core, they are soluble and usually secreted into the extracellular space. Secreted GAGs can then be isolated and used as probes for understanding the GAG biosynthesis as well as for investigations of pathogen-GAG interactions. Quantities and structures of the primed GAGs are dependent on both aglycone and cell type.^16–25^ Since these β-d-xylopyranosides are substrates for β4GalT7, they provide a starting point for the design of inhibitors against this enzyme.

A decade ago, the crystal structures of β4GalT7, in both an open and a closed conformation, were published.^26^ Inspection of the structure and comparison with conserved motifs in other galactosyltransferases strongly suggest that β4GalT7 functions in a similar manner as β4GalT1 (Figure 1c).^27–29^ First, the enzyme binds a Mn^2+^ ion, followed by complexation of a UDP-Gal donor. The diphosphate coordinates to the metal ion and generates a conformational change from an open to a closed state. During this change, His259 coordinates to the Mn^2+^ ion, which seemingly causes the protein backbone to shift and form the acceptor pocket. This allows the binding of the acceptor substrate, which then undergoes an S_N_2 reaction to form the galactosylated product.

The thermodynamic changes of the donor substrate binding to β4GalT7 have been investigated.^30^ The most important binding moieties in the donor substrate for β4GalT7 are the uracil and the β-phosphate groups (Figure 2a). The β-phosphate interacts through the Mn^2+^ via a DXD motif, which is highly conserved in galactosyl transferases that have the GT-A fold.^31,32^ The α-phosphate has a minor influence on binding. However, hydrogen bonding patterns are important for binding as seen for the *Drosophila* β4GalT7 by interactions between Tyr177 and the β-phosphate group of the donor and for specificity as identified by hydrogen bonding of the hydroxyl groups HO2 and HO3 in galactose to Asp145 and HO4 in galactose to Glu210 in β4GalT7.^26^ Furthermore, *p*-nitrophenyl β-d-xyloside binds to β4GalT7 in the presence of UDP, which further implies that the galactose part of UDP-Gal is less important to form the closed conformation.^26^

**Figure 2:**
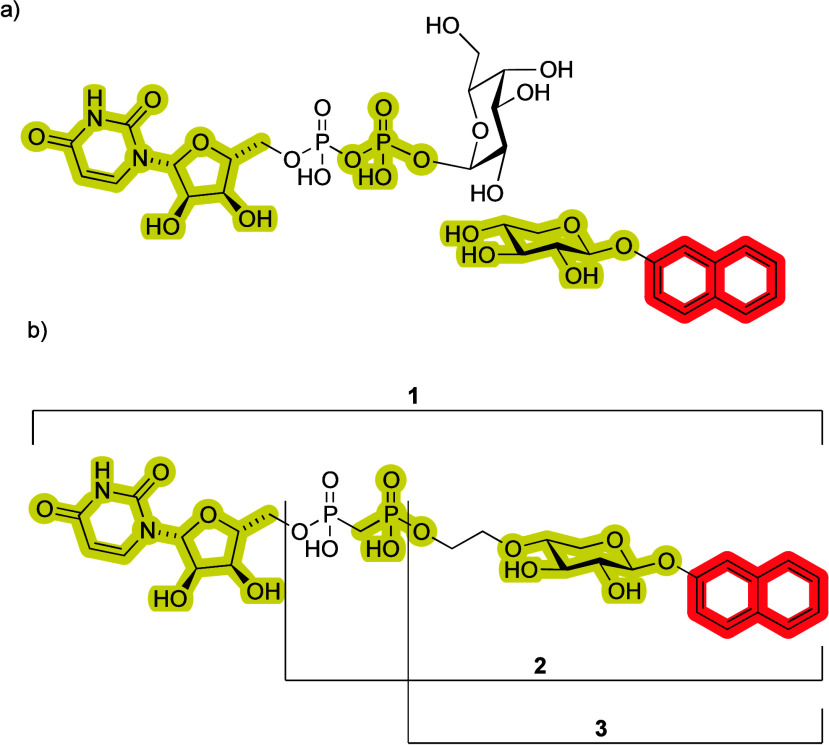
(a) Relative importance of the binding motifs in β4GalT7. The most important in the donor substrate is the uracil and the β-phosphate. Furthermore, the **XylNap** moiety is important for effective binding, whereas the Gal unit is less important. (b) Target molecules of this study.

One interesting approach in GT inhibitor design would be the use of chimeras of the donor and the acceptor, i.e., a transition state analog approach, which hopefully would provide both specificity and strong binding. We thus hypothesize that selective inhibitors of β4GalT7 can be formed by a combination of parts of the donor and the acceptor.

In this study, we investigate new potential inhibitors of the GAG biosynthesis and evaluate the interactions between β4GalT7 and several pentosides in the presence of different UDP-donors using STD NMR spectroscopy.

## Results and Discussion

### NMR Studies

Inspired by the above-mentioned findings, we used ^1^H and STD NMR to evaluate the binding of several previously synthesized naphthopentosides, in the presence of donors.^11,33,34^ Thus, we investigated if and how binding to β4GalT7 took place and whether product formation could be observed.

A selection of acceptor xylosides (**XylBn**, **XylMe**, **XylNap**, **XylNapOH**) and analogs in which the stereochemistry at C3 or C4 had been inverted (**RibNap** and **AraNap**, respectively) were chosen (Table 1). UDP-Gal, the natural donor, and UDP-Glc as well as UDP-GalNAc were chosen as donors. ^1^H NMR experiments acquired with XylNap:UDP-Gal:β4GalT7 and XylNapOH:UDP-Gal:β4GalT7 mixtures at different time points showed GalXylNap and GalXylNapOH product formation, consistent with previous studies using biochemical assays.^35^ Moreover, **XylMe**, a **XylNap** competitor in the galactosylation reaction, and **XylBn**, which can prime GAGs in animal cells, were suggested to be galactosylated.^35,36^ ^1^H NMR experiments of acceptor xylosides and analogs in the presence of β4GalT7 and either UDP-Glc or UDP-GalNAc did not show product formation (data not shown).

**Table 1:**
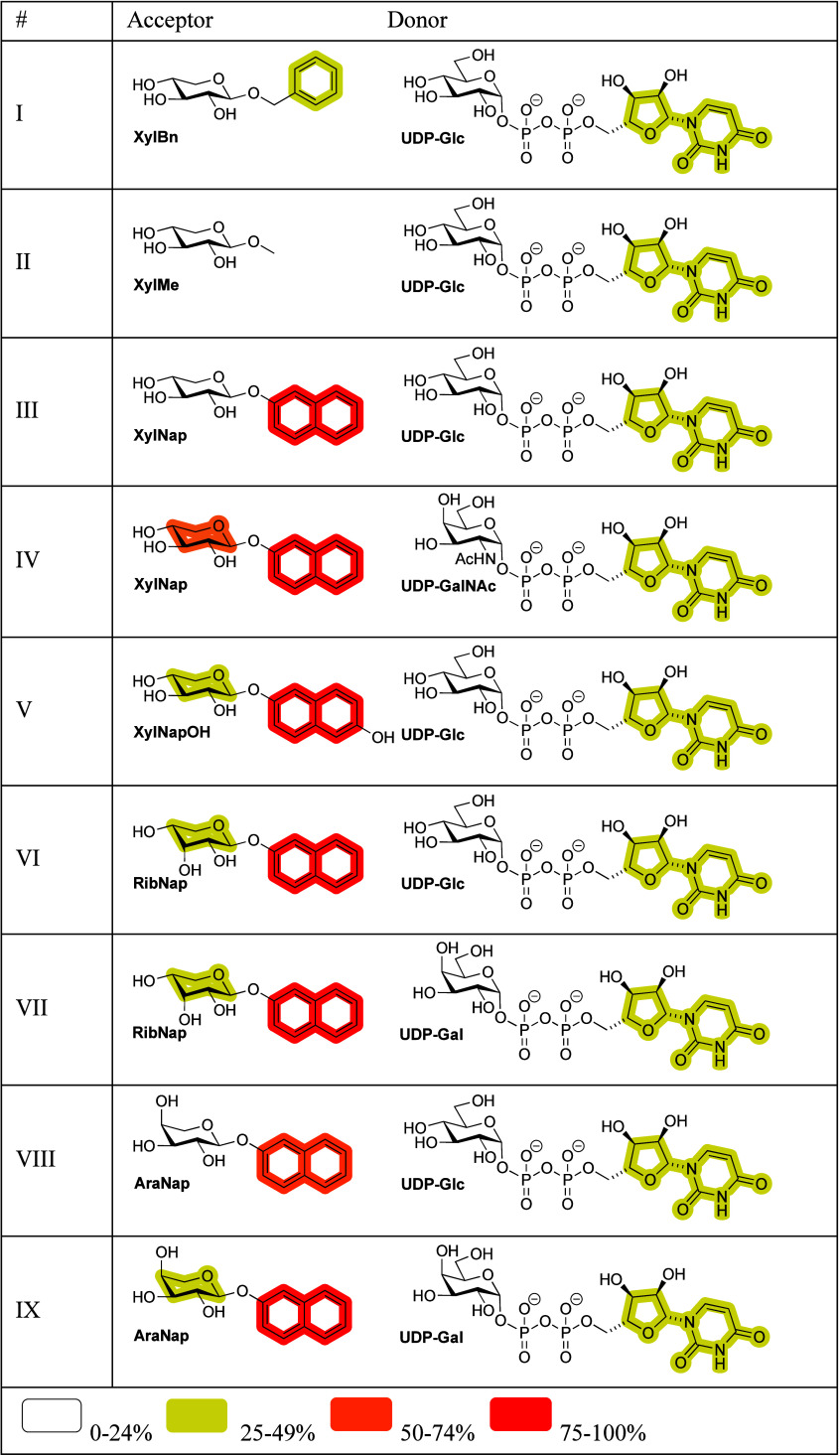
STD NMR Analysis of Xylosides and Analogues Thereof in Conjunction with UDP-Hexoses and β4GalT7^a^

a The tested combinations of ligands are denoted by I–IX. The average STD percentage per entity of acceptor molecule (pentose and aglycone) and donor molecule (hexose, Ribf, and uracil) are grouped into four categories based on the relative intensity of the STD signals.

To further examine the binding events of β4GalT7, we tested combinations of ligands denoted I–IX (Table 1). For the non-natural acceptors with the *ribo*- and *arabino*-configuration, product formation was limited or insignificant, with only trace amounts suggested to be formed.

We utilized STD NMR to investigate the binding of ligands to β4GalT7.^37,38^ The signal with the largest STD effect was normalized to 100%. An average STD percentage was calculated for each entity using resolved ^1^H resonances and grouped into four categories based on intensity levels (Table 1). The same binding epitope was observed for all donors in I–IX, with an intermediate STD effect for the uracil moiety. For the acceptors, the 2-naphthyl moiety was the entity with the most considerable STD effects, with an average of >75% for all the Nap-containing systems except for entry VIII, where it still was above 50%. The benzyl group of **XylBn** showed an intermediate STD effect, while the response was low for the methyl group of **XylMe** (entries I and II, respectively)

For the non-natural UDP-hexoses (in relation to β4GalT7), binding occurred with intermediate and similar STD effects seen in all nine cases for the nucleotide portion of the molecules (Table 1), whereas the hexose moieties showed weak STD effects overall. For the nonproductive acceptors, where **AraNap** has also been shown to be a competitor to the galactosylation reaction, STD effects of the glycan entity were generally of intermediate-to-low intensity.^35^

It is noteworthy that the STD effect of the xylose moiety was moderately stronger when the hexose has the *galacto*-configuration (entry IV), and that β4GalT7 tolerates an *N*-acetyl group at C2 of the sugar residue, in comparison to when it has the *gluco*-configuration (entry III). Interestingly, all non-natural combinations of acceptors and donors, mainly with a change in configuration at a single stereogenic center, bind to β4GalT7. However, efficient glycosylation reaction is prevented, which emphasizes that small differences in substrates can significantly impact the binding specificity and functioning of galactosyltransferases.

### Inhibitor Design

We have previously investigated a range of naphthoxylosides as inhibitors for β4GalT7.^11,39^ In general, modification of the xylose moiety was not tolerated well by the enzyme, as observed by other groups.^40^ These findings, together with the STD NMR data above and the importance of the β-phosphate group for the interaction between donor and β4GalT7, made us take a different approach. Specifically, we envisioned target **1**, as an elongation of the **XylNap** scaffold with a hydrolytically stable UDP analog. Diphosphates are susceptible to both hydrolysis and enzymatic cleavage and methylene bisphosphonates have been introduced as stable bioisosters.^41–43^ This chimera could potentially form a strong transition state inhibitor of β4GalT7 (Figure 2b). By binding to the open conformation of the enzyme, the chimera could be captured in the conformational switch from open to closed conformation and thereby leveraging the combined interactions from the uridine and the naphthyl moiety with the protein. In addition, we decided to search for a minimum inhibitory epitope by testing the truncated analogs **2** and **3.**

### Synthesis

One option to introduce a methylene bisphosphonate is to prepare the tetraester of a bisphosphonate followed by selective deprotection, chlorination, and subsequent condensation.^44–48^ This route is significantly more convenient compared to a carbanionic approach,^37^ where extreme care has to be taken due to the toxicity of some intermediates, for instance, methyl phosphonic dichloride (LD_50_ 2.4 ppm by inhalation (rat)).^49,50^ Other methods are based on combined phosphoramidite-phosphodiester reagents that can significantly improve the yields of these reactions.^43^

The known xyloside **5** ^26^ was alkylated with an α-haloester to give **6**, giving linker fragment **7** after ester reduction.^11^ The condensation of the three fragments then proceeded in a one-pot fashion by sequential addition. Commercially available **8** was first added to methylene bis­(phosphonic dichloride) in triethyl phosphate, followed by **7** upon consumption of **8**. When the condensation was deemed complete, AcOH (aq.) was added to hydrolyze the acetals to give the target compound **1**. Next, **2** was synthesized by a similar procedure without adding the uridine moiety. Since the reaction was performed in a one-pot manner, we observed several undesired side-products, mainly the symmetric phosphonates. Finally, **3** was obtained via acetal hydrolysis of **7**. Compound **4**, as detailed below, was synthesized by hydrolysis of **2** using HCl under microwave heating. The synthesis of compounds **1**, **2**, and **4** proved to be inconsistent, particularly with **1** showing a high tendency to degrade under acidic conditions, in addition to formation of symmetrical products. To improve the synthesis of **1**, we explored several alternative strategies, including the use of tetrabutylammonium salts of bisphosphonic acid in combination with tosylated uridine and xyloside derivatives. Additionally, we employed peptide coupling methodologies such as DCC, EDC with HOBt, and HATU in DMF or acetonitrile. However, all these approaches were unsuccessful. Based on our experience, we do not recommend the synthesis of these types of compounds via these routes, as their acid sensitivity appears to be a significant limiting factor.

### β4GalT7 Inhibition Assay and Cellular Studies

To investigate the inhibitory potential of the three potential inhibitors **1**–**3**, we used a modified approach to our previously published β4GalT7 assay.^51^ In short, the formation of the GalXylNap disaccharide was analyzed by reversed-phase chromatography, with addition of potential inhibitors. We thus observed an apparent decrease in product formation in the presence of **2** (72% inhibition compared to control, see Supporting Information, Figure S1), whereas **1** and **3** did not inhibit the enzyme. Previous results have shown up to 64% inhibition by a fluorinated xyloside at 2 mM, in comparison to this experiment where we used 1 mM of the compound.^11^

When compound **2** was tested in a concentration-dependent assay we experienced complications with reproducibility (Supporting Information, Figure S2). The phosphonate group is acidic, which significantly lowered the pH in the stock solution, leading to decomposition of compound **2**. Consequently, we detected free 2-naphthol that indicated acidic hydrolysis of the glycosidic bond. Furthermore, compound **2** seemed to form a precipitate with Mn^2+^ in the buffer. To minimize acidic hydrolysis, we increased the buffer strength of the stock solution. When we used this new stock solution, which contained only **2** and no hydrolysis products, the inhibition was abolished (Supporting Information, Figure S3). We therefore hypothesized that the actual inhibitor was the hydrolysis product **4**, rather than compound **2.**

To explore this observation, we hydrolyzed **2** using HCl under microwave heating (Scheme 1) to the corresponding hydrolysis products (i.e., **4** and 2-naphthol), and evaluated them as inhibitors (Figure 3a).

**Scheme 1:**
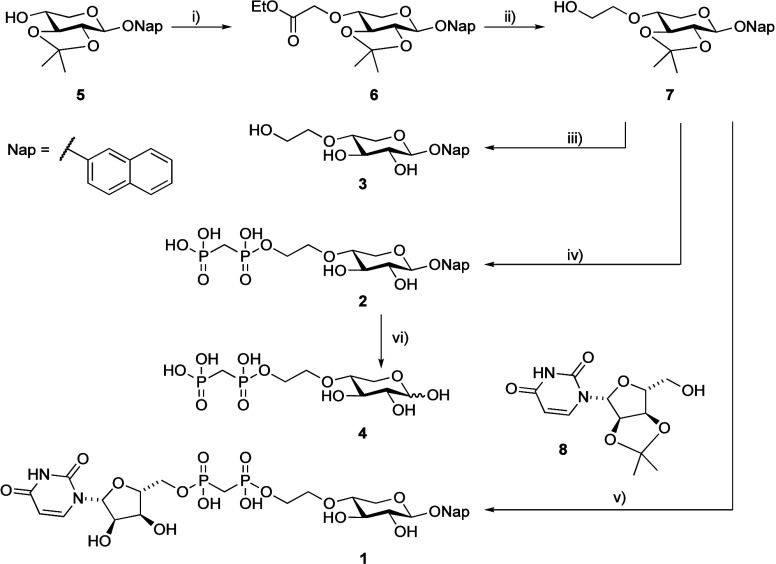
Synthesis of the Target Molecules^a^

a Reagents and conditions: (i) NaH (60% disp. in mineral oil), ethyl iodoacetate, THF, 0 °C, 71%; (ii) LiBH_4_, THF, 0 °C, 78%; (iii) 70% AcOH, 61%; (iv) CH_2_(P­(O)­Cl_2_)_2_, PO­(OEt)_3_, DIPEA, 0 °C to r.t., then 70% AcOH, 55 °C, 8%; (v) CH_2_(P­(O)­Cl_2_)_2_, PO­(OEt)_3_, DIPEA, 0 °C to r.t., then 70% AcOH, 55 °C, 1%; (vi) 1 M HCl, MW 95 °C, 67%.

**Figure 3:**
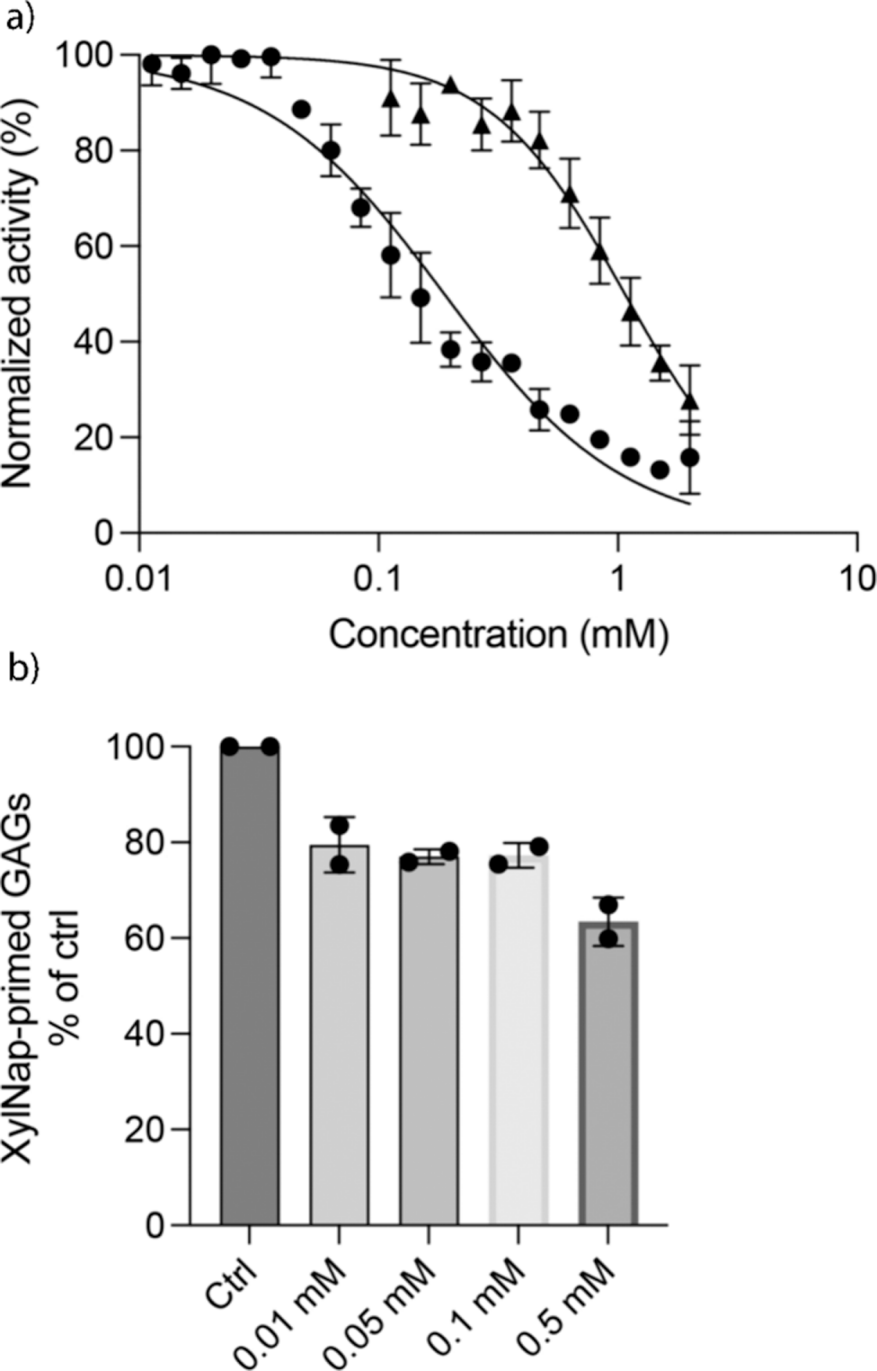
(a) Concentration-dependent assays of **4** (filled circles) and 2-naphthol (filled triangles). (b) HEK293 cells were treated with **XylNap** in the presence of various concentrations of **4**, after which the xyloside-primed GAG chains were isolated and analyzed by SEC-fluorescence.

Both **4** (anomeric mixture β:α 1.8:1) and 2-naphthol display concentration-dependent inhibition of β4GalT7. For **4**, we calculated an IC_50_ of 188 μM. As expected from the STD NMR experiments, and verified by the assay, there seem to be strong interactions between 2-naphthol and β4GalT7. The lack of inhibition by the full-length compound **1** could then potentially be explained by the 2-naphthyl moiety forming strong interactions with the outer parts of the enzyme and thus disrupting the interactions in the UDP site.

As a proof of principle and to investigate if the inhibitory effects could be transferred to cells, we treated HEK293 cells with increasing concentrations of **4** in the presence of **XylNap**. The **XylNap**-primed GAGs were purified by anion exchange chromatography and analyzed by size-exclusion chromatography (SEC)-fluorescence (Figure 3b). The results indicated a weak inhibitory effect by **4**, showing that the compound does act as a GAG biosynthesis inhibitor in cells.

## Conclusions

In our search for new inhibitors of GAG biosynthesis, we used ^1^H and STD NMR to study the binding between β4GalT7 and several pentosides in the presence of UDP-donors to test the enzyme binding and glycosylation ability. These investigations verified the glycosylation specificity of β4GalT7, and we found that naphthalene and uridine moieties significantly contributed to the acceptor and donor binding, respectively. Based on these findings, we investigated the binding of donor–acceptor chimeras by extending the naphthoxyloside with variations of a uridine-bisphosphonate-linker moiety. Using a one-pot approach, we synthesized the different targets without the isolation of intermediates. One compound, **2**, displayed a strong inhibitory effect. However, a close investigation revealed that its hydrolysis product, **4**, was the actual inhibitor with an IC_50_ of 188 μM. Intriguingly, it seems like **2** is too short to simultaneously accommodate both bisphosphonate and naphthyl binding. These results suggest that bisphosphonate-xyloside constructs could be a new class of GAG biosynthesis inhibitors.

## Materials and Methods

### Synthesis

All moisture- and air-sensitive reactions were carried out under an atmosphere of dry nitrogen using oven-dried glassware. All solvents were dried using an MBRAUN SPS-800 Solvent purification system before use unless otherwise stated. Purchased reagents were used without further purification. Chromatographic separations were performed on Matrex silica gel (25–70 μm). Thin-layer chromatography was performed on precoated TLC aluminum plates with silica gel 60 F_254_ 0.25 mm (Merck). Spots were visualized with UV light or by charring with an ethanolic anisaldehyde solution. Biotage Isolute phase separators were used for drying of combined organic layers. Preparative chromatography was performed on Biotage Isolera One flash purification system using Biotage SNAP KP-Sil silica cartridges. Optical rotations were measured on a Bellingham and Stanley model ADP450 polarimeter and are reported as [α]_D_^*T*^ (*c* = g/100 mL), D indicate the sodium D line (589 nm), and *T* indicates the temperature (°C). NMR spectra for characterization of synthesized compounds were recorded at ambient temperatures on a Bruker Avance II at 400 MHz (^1^H) and 100 MHz (^13^C) or a Bruker Ascend at 500 MHz (^1^H) and 125 MHz (^13^C) and assigned using 2D methods (COSY, HMQC). Chemical shifts are reported in ppm, with reference to residual solvent peaks (δ_H_ CDCl_3_ = 7.26 ppm, CD_3_OD = 3.31 ppm) and solvent signals (δ_C_ CDCl_3_ = 77.0 ppm, CD_3_OD = 49.0 ppm). Coupling constant values are given in Hz. Mass spectra were recorded on Waters XEVO G2 (Positive ESI).

#### 2-Naphthyl 4-*O*-(2-(uridine-5-α,β-methylene-diphosphoryl)­hydroxyethyl)-β-d-xylopyranoside (**1**)

Methylenebis­(phosphonic dichloride) (48 mg, 0.194 mmol) was dissolved in freshly distilled triethyl phosphate (4 mL) while stirring, and cooled to 0 °C under Ar­(g) atmosphere. DIPEA (169 μL, 0.972 mmol) was added, followed by **8** (46 mg, 0.162 mmol). The reaction was allowed to reach r.t. Upon consumption of **8**, the mixture was cooled to 0 °C and **7** (58 mg, 0,162 mmol) in dry DCM was added dropwise. Upon consumption of **7**, the mixture was acidified with 70% AcOH and heated to 55 °C. When the reaction was deemed complete, the mixture was neutralized with NaHCO_3_ (sat. aq), and the volatiles removed by rotary evaporation. The crude was purified by preparative HPLC, and lyophilized to give **1** (1.5 mg, 0.0021 mmol, 1.3%) as an amorphous white solid. [α]_D_^20^ = −2.2 (*c* = 0.46 g cm^–3^ in CD_3_OD). ^1^H NMR (400 MHz, CD_3_OD) δ 7.94 (d, *J* = 8.1 Hz, 1H), 7.81–7.77 (m, 3H), 7.48–7.40 (m, 2H), 7.35 (dd, *J* = 8.1, 6.7 Hz, 1H), 7.26 (dd, *J* = 9.0, 2.5 Hz, 1H), 5.95 (d, *J* = 4.9 Hz, 1H), 5.80 (d, *J* = 8.1 Hz, 1H), 5.04 (d, *J* = 7.3 Hz, 1H), 4.32–4.10 (m, 8H), 3.89 (d, *J* = 3.5 Hz, 2H), 3.65–3.43 (m, 4H), 2.51 (t, *J* = 20.8 Hz, 2H). ^13^C NMR (125 MHz, CD_3_OD) δ 166.22, 156.63, 152.54, 142.78, 135.85, 131.30, 130.34, 128.60, 128.17, 127.38, 125.26, 120.02, 111.92, 103.13, 102.76, 90.18, 84.58, 79.97, 76.81, 75.36, 74.73, 71.48, 71.21, 66.59, 65.72, 64.77, 17.43. ^31^P NMR (162 MHz, CD_3_OD) δ 19.33, 17.73. HRMS (*m*/*z*): [M]^+^ calcd for C_27_H_34_N_2_O_16_P_2_Na: 727.1281; found: 727.1290.

#### 2-Naphthyl 4-*O*-(2-(methylene-diphosphoryl)­hydroxyethyl)-β-d-xylopyranoside (**2**)

This was prepared similarly as described for **1** from **7** (195 mg, 0.541 mmol), to give **2** (20 mg, 0,042 mmol, 8%) as an amorphous white solid. [α]^D^_20_ = −20.2 (*c* = 0.99 g cm^–3^ in CD_3_OD). ^1^H NMR (400 MHz, CD_3_OD) δ 7.81–7.76 (m, 3H), 7.47–7.39 (m, 2H), 7.35 (ddd, *J* = 8.1, 6.9, 1.3 Hz, 1H), 7.26 (dd, *J* = 8.9, 2.5 Hz, 1H), 5.03 (d, *J* = 7.2 Hz, 1H), 4.26–4.14 (m, 3H), 3.90 (q, *J* = 4.0 Hz, 2H), 3.66–3.43 (m, 4H), 2.49 (t, *J* = 21.0 Hz, 2H). ^13^C NMR (100 MHz, CD_3_OD) δ 155.22, 134.42, 129.90, 128.94, 127.21, 126.71, 125.99, 123.86, 118.60, 110.52, 101.40, 78.52, 75.44, 73.32, 69.98, 65.34, 63.34, 26.19. ^31^P NMR (162 MHz, CD_3_OD) δ 19.88, 17.07. HRMS (*m*/*z*): [M]^+^ calcd for C_18_H_24_O_11_P_2_Na: 501.0692; found: 501.0693.

#### 2-Naphthyl 4-*O*-(2-hydroxyethyl)-2,3-*O*-isopropylidene-β-d-xylopyranoside (**3**)

Compound **7** (64 mg, 0.178 mmol) was dissolved in 70% AcOH (2 mL), and heated to 40 °C. Upon completion, the mixture was neutralized with NaHCO_3_ (sat. aq), and the volatiles removed by rotary evaporation. The crude was purified by preparative HPLC, and lyophilized to give **3** (35 mg, 0.109 mmol, 61%) as an amorphous white solid. [α]^D^_20_ = −5.5 (*c* = 1.00 g cm^–3^ in CD3OD). ^1^H NMR (400 MHz, CD_3_OD) δ 7.81–7.74 (m, 3H), 7.46–7.40 (m, 2H), 7.35 (ddd, *J* = 8.1, 6.9, 1.3 Hz, 1H), 7.26 (dd, *J* = 8.9, 2.5 Hz, 1H), 5.03 (d, *J* = 7.2 Hz, 1H), 4.21–4.09 (m, 1H), 3.80–3.66 (m, 4H), 3.63–3.50 (m, 2H), 3.49–3.40 (m, 2H). ^13^C NMR (100 MHz, CD_3_OD) δ 156.63, 135.82, 131.31, 130.36, 128.62, 128.11, 127.40, 125.27, 120.00, 111.92, 102.82, 79.79, 76.85, 74.69, 73.54, 64.72, 62.63. HRMS (*m*/*z*): [M]^+^ calcd for C_17_H_20_O_6_Na: 363.1158; found: 363.1155.

#### 4-*O*-(2-(Methylene-diphosphoryl)­hydroxyethyl)-α/β-d-xylopyranose (**4**)

Compound **2** (15 mg, 0,031 mmol) was dissolved in **1** mL H_2_O and acidified with 1 M HCl. The mixture was heated using microwave irradiation at 95 °C for 1 h. Upon completion, the reaction was neutralized using 2% NH_3_ solution. The mixture was purified by preparative HPLC, and lyophilized to give **4** (anomeric mixture β:α 1.8:1, 7 mg, 0,021 mmol, 67%) as an amorphous solid. ^1^H NMR (400 MHz, D_2_O) δ 5.10 (d, *J* = 3.7 Hz, 1H), 4.49 (d, *J* = 7.9 Hz, 1.8H), 4.12–3.94 (m, 13.2H), 3.88–3.75 (m, 12.4H), 3.72–3.56 (m, 2.8H), 3.52–3.37 (m, 6.7H), 3.32–3.13 (m, 2.5H), 2.46–2.27 (m, 12H). ^13^C NMR (101 MHz, D_2_O) δ 96.49, 92.00, 78.04, 74.79, 73.91, 71.77, 71.34, 70.32, 64.59, 63.11, 58.91, 26.09 (t, *J* = 130.1 Hz). ^31^P NMR (162 MHz, D_2_O) δ 19.01, 18.19. HRMS (*m*/*z*): [M]^+^ calcd for C_8_H_18_O_11_P_2_Na: 375.0222; found: 375.0233.

#### 2-Naphthyl 4-*O*-(2-ethoxy-2-oxoethyl)-2,3-*O*-isopropylidene-β-d-xylopyranoside (**6**)

Compound **5** (926 mg, 2.929 mmol) was dissolved in dry THF (10 mL) while stirring and cooled to 0 °C. NaH (60% suspension in mineral oil, 147 mg, 3.661 mmol) was added in two portions. After 15 min, the reaction was allowed to reach r.t. After 1 h, ethyl iodoacetate (693 μL, 5.858 mmol) was added dropwise. Upon completion, the reaction was diluted with EtOH, alkalinized with Et_3_N and volatiles removed by rotary evaporation. Purification by flash chromatography (Petroleum ether: EtOAc 5% to 33% containing 1% Et_3_N) gave **6** (838 mg, 2.082 mmol, 71%) as a clear oil. [α]^D^_20_ = −25.2 (*c* = 0.95 g cm^–3^ in CD_3_OD). ^1^H NMR (400 MHz, CD_3_OD) δ 7.81–7.77 (m, 3H), 7.51–7.44 (m, 2H), 7.40 (ddd, *J* = 8.1, 6.9, 1.3 Hz, 1H), 7.29 (dd, *J* = 8.9, 2.5 Hz, 1H), 5.55 (d, *J* = 6.6 Hz, 1H), 4.27 (dd, *J* = 12.4, 5.1 Hz, 1H), 3.96–3.72 (m, 7H), 3.64 (dd, *J* = 12.4, 5.5 Hz, 1H), 1.57–1.55 (m, 3H), 1.53 (d, *J* = 0.7 Hz, 3H). ^13^C NMR (101 MHz, CDCl_3_) δ 154.42, 134.36, 130.06, 129.64, 127.79, 127.32, 126.57, 124.54, 119.18, 112.60, 111.27, 99.64, 80.28, 77.36, 76.67, 71.59, 66.06, 62.27, 27.07, 26.87. HRMS (*m*/*z*): [M]^+^ calcd for C_22_H_24_ONa: 383.1471; found: 383.1465.

#### 2-Naphthyl 4-*O*-(2-hydroxyethyl)-2,3-*O*-isopropylidene-β-d-xylopyranoside (**7**)

Compound **6** (553 mg, 1.374 mmol) was dissolved in dry THF (14 mL) while stirring and cooled to 0 °C. LiBH_4_ (60 mg, 2.745 mmol) was added. Upon completion, the reaction was diluted with MeOH, and volatiles removed by rotary evaporation. Purification by flash chromatography (Petroleum ether: EtOAc 50% to 100% containing 1% Et_3_N) gave **7** (388 mg, 1.077 mmol, 78%) as a clear oil. [α]^D^_20_ = −50.5 (*c* = 0.91g cm^–3^ in CD_3_OD). ^1^H NMR (400 MHz, CD_3_OD) δ 7.81–7.76 (m, 3H), 7.48–7.40 (m, 2H), 7.36 (ddd, *J* = 8.1, 6.9, 1.3 Hz, 1H), 7.23 (dd, *J* = 8.9, 2.5 Hz, 1H), 5.55 (d, *J* = 7.2 Hz, 1H), 4.28–4.19 (m, 3H), 3.96 (ddd, *J* = 8.4, 5.9, 5.2 Hz, 1H), 3.87 (dd, *J* = 9.7, 8.5 Hz, 1H), 3.71 (dd, *J* = 9.7, 7.2 Hz, 1H), 3.67 (dd, *J* = 12.3, 6.0 Hz, 1H), 1.49 (s, 3H), 1.47 (s, 3H), 1.29 (t, *J* = 7.1 Hz, 5H). ^13^C NMR (101 MHz, CDCl_3_) δ 170.33, 154.42, 134.35, 130.07, 129.62, 127.78, 127.34, 126.55, 124.53, 119.18, 112.63, 111.31, 99.64, 80.14, 76.68, 67.30, 65.89, 61.16, 27.08, 26.80, 14.36. HRMS (*m*/*z*): [M]^+^ calcd for C_22_H_26_O_7_Na: 425.1576; found: 425.1573

### NMR Spectroscopy Experiments for Interaction Studies

The donors and acceptor samples were prepared as 10 mM in phosphate buffer in D_2_O (10 mM pD 6.6, 150 mM NaCl, 15 mM MgCl_2_) containing 5% DMSO-*d*_6_ and transferred to 5 mm NMR tubes. NMR experiments were carried out at 8 °C where the temperature had been calibrated with a sample of neat deuterated methanol.^52^ ^1^H and ^13^C NMR chemical shifts were referenced to an internal 3-trimethylsilyl-(2,2,3,3-^2^H_4_)-propanoate (TSP, δ_H_ 0.00) and 1,4-dioxane in D_2_O (δ_C_ 67.40). For assignments of NMR resonances standard one-dimensional ^1^H NMR, two-dimensional ^1^H,^13^C-HSQC, ^1^H,^1^H-TOCSY (mixing times of 30 and 60 ms), ^1^H,^13^C-HMBC and ^1^H,^1^H-NOESY (mixing time 300 ms) experiments were carried out on a 500 MHz Bruker AVANCE spectrometer equipped with a TCI Z-Gradient cryoprobe. Saturation transfer difference (STD)^37,38^ NMR experiments of donors and acceptors (I–IX) in the presence of βGalT7 were acquired with a 1:200:200 protein:donor:acceptor ratio (5 μM, 1 mM and 1 mM, respectively). On- and off-resonance frequencies were set at 0.66 and 60 ppm, respectively, employing a 2 s irradiation time. STD NMR experiments were acquired with 1024 scans and 16384 data points. For analysis of the NMR spectra, the CARA software was used.^53^

### β4GalT7 Inhibition Assay

Potential inhibitors of β4GalT7 were screened in an enzymatic assay as previously described.^11^ In short, β4GalT7 (50 ng) together with UDP-Gal (1 mM final concentration) and various concentrations of xylosides were added to 96-well polypropylene plates in a final volume of 50 μL MES buffer (20 mM, pH 6.2) supplemented with MnCl_2_ (10 mM). Incubations were performed at 37 °C for 30 min and the reaction was stopped by cooling at 4 °C and addition of HPLC eluent (70% NH_4_OAc (60 mM, pH 5.6)–30% CH_3_CN (v/v)) before HPLC analysis on an ACE C8, 3 μm, 4.6 × 100 mm (ACE) HPLC column.

### Cellular Studies

HEK293 cells were cultured in a humidified CO_2_ (8%) incubator at 37 °C to approximately 70% confluence in DMEM/F-12/GlutaMAX (Thermo Fisher Scientific) supplemented with 10% FBS (Thermo Fisher Scientific), 100 units mL^–1^ penicillin and 100 μg mL^–1^ streptomycin (Sigma-Aldrich). For GAG-inhibition studies, the growth medium was aspirated and **4** was added into OptiPRO SFM medium (Thermo Fisher Scientific) to a final concentration of 0.01, 0.05, 0.1, and 0.5 mM from 100× stock solutions in DMSO. Immediately after, **XylNap** was added to a final concentration of 100 μM from a 50 mM stock solution in DMSO. The cells were incubated for 24 h and GAGs were purified from medium samples using an anion exchange DE52 diethylaminoethyl cellulose resin (Sigma-Aldrich) and analyzed by fluorescence detection size-exclusion chromatography (FSEC) on an AdvanceBio SEC column (Agilent). The relative amounts of GAGs primed was quantified by area integration of the XylNap-GAG peak and subsequent normalization to the control sample without **4**.

## Supplementary Material



## Notes

Additional experimental data and NMR spectra (PDF)

## #

The University of Hong Kong, G02-G04, CYM Chemistry Building, Pokfulam Road, Hong Kong (P.B.)
