# Supplementary material for: Design of Glycosyltransferase Inhibitors: Targeting the Biosynthesis of Glycosaminoglycans by Phosphonate-Xyloside
Source: ACS Omega. 2025 Nov 17;10(47):57210–8. doi: 10.1021/acsomega.5c06840 (PMC12676349; doi:10.1021/acsomega.5c06840)
Supplement: Supplementary file 1 [file ao5c06840_si_001.pdf]

# Design of glycosyltransferase inhibitors: targeting the biosynthesis of glycosaminoglycans by phosphonate-xyloside

Daniel Willén,[a] Hanna Malmquist,[a] Pilar Blasco,[b#] Joachim Björklund,[a] Roberto Mastio,[a] Sophie Manner,[a] Göran Widmalm,[b] Emil Tykesson,[c] Ulf Ellervik*[a,c]

[a] Lund University, Department of Chemistry, Centre for Analysis and Synthesis, P.O. Box 124, SE-221 00 Lund, Sweden.
[b] Stockholm University, Arrhenius Laboratory, Department of Organic Chemistry, SE-106 91 Stockholm, Sweden.
[c] Lund University, Department of Experimental Medical Science, P.O. Box 117, SE-221 00 Lund, Sweden.

* Email: Ulf Ellervik, ulf.ellervik@chem.lu.se

*Keywords: β4GalT7, xyloside, glycosaminoglycan, UDP-Gal, phosphonate*

**ABSTRACT:** β-1,4-galactosyltransferase 7 (β4GalT7) is a key enzyme in the biosynthesis of glycosaminoglycans (GAG) that transfers the first galactose unit to xylose in the linker region. Searching for new inhibitors of the GAG biosynthesis, we used saturation transfer difference (STD) NMR spectroscopy to evaluate the binding interactions between β4GalT7 and several pentosides in the presence of UDP-donors. These investigations verified the glycosylation specificity of β4GalT7 and revealed that the naphthalene and the uridine moieties were significant contributors to the binding of the acceptor and the donor, respectively, while the galactose part was less important. Based on these findings, we set out to investigate conjugates of UDP and naphthoxylosides to function as transition state analogs. These compounds were synthesized using a one-pot procedure and tested as inhibitors in a β4GalT7 assay. Interestingly, one truncated analog, a bisphosphonate-xyloside construct, showed a significant inhibition ($IC_{50}$: 188 μM). These findings open for the design of a new class of inhibitors of the GAG biosynthesis.

## Supporting information


INHIBITION ASSAY (FIGURE S1-S3) .......... 3

$^{1}$H-, $^{13}$C-, AND $^{31}$P-NMR OF COMPOUND 1 (FIGURE S4-S6) .......... 5

$^{1}$H-, $^{13}$C-, AND $^{31}$P-NMR OF COMPOUND 2 (FIGURE S7-S9) .......... 7

$^{1}$H- AND $^{13}$C-NMR OF COMPOUND 3 (FIGURE S10-S11) .......... 9

$^{1}$H-,$^{13}$C-, AND $^{31}$P-NMR OF COMPOUND 4 (FIGURE S12-S14) .......... 10

$^{1}$H- AND $^{13}$C-NMR OF COMPOUND 6 (FIGURE S15-S16) .......... 12

$^{1}$H- AND $^{13}$C-NMR OF COMPOUND 7 (FIGURE S17-S18) .......... 13

STD- AND $^{1}$H-NMR OF COMBINATIONS I – IX OF LIGANDS (FIGURE S19-27) .......... 14

## Inhibition assay (Figure S1-S3)

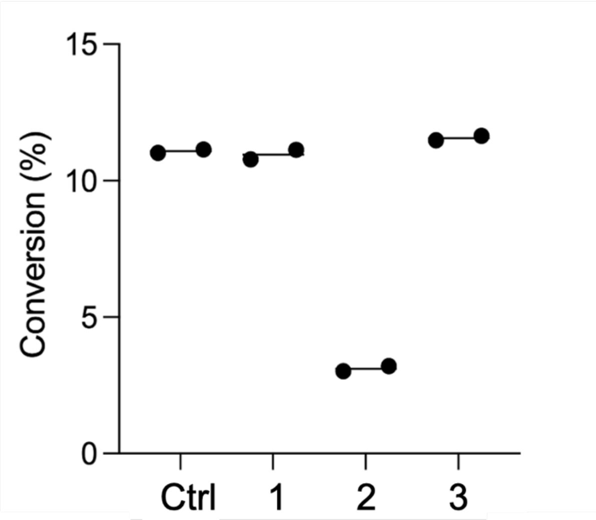


**Figure S1 –** The formation of the GalXylNap disaccharide was analyzed by reversed-phase chromatography, with addition of potential inhibitors. We thus observed an apparent decrease in product formation in the presence of **2** (72% inhibition compared to control), whereas **1** and **3** did not inhibit the enzyme.

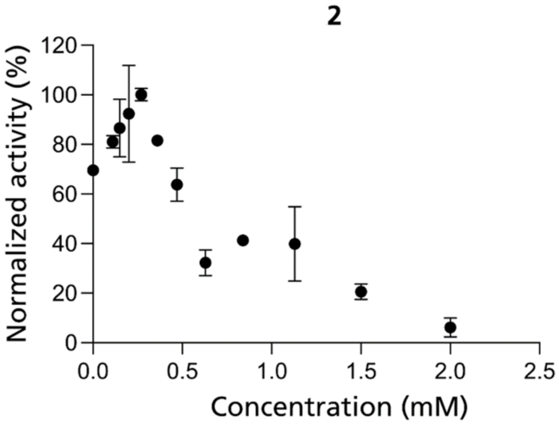


**Figure S2** – **2** displaying concentration-dependent inhibition (early data)

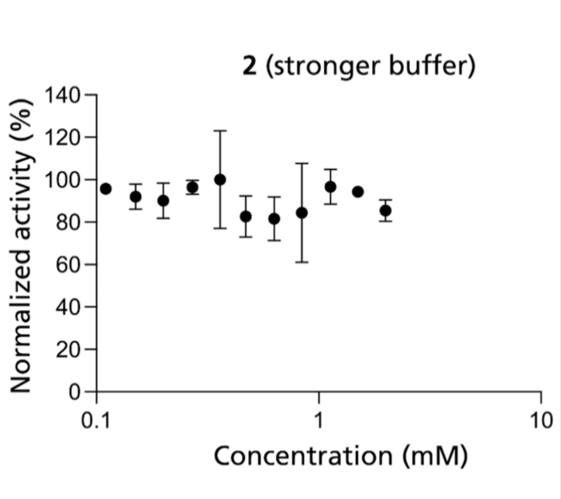


**Figure S3** – Attempt to replicate findings with stronger buffer conditions

## $^{1}$H-, $^{13}$C-, and $^{31}$P-NMR of compound 1 (Figure S4-S6)

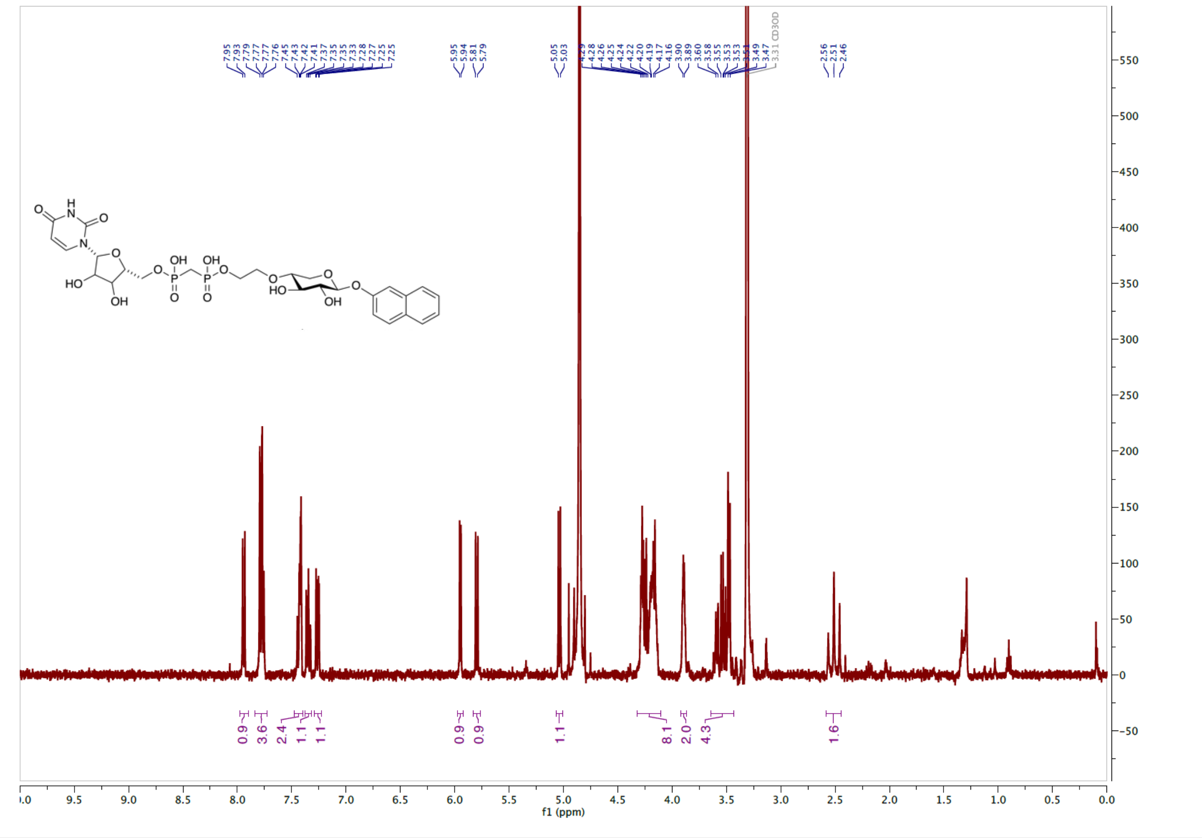

**Figure S4** – $^{1}$H-spectra of compound **1**

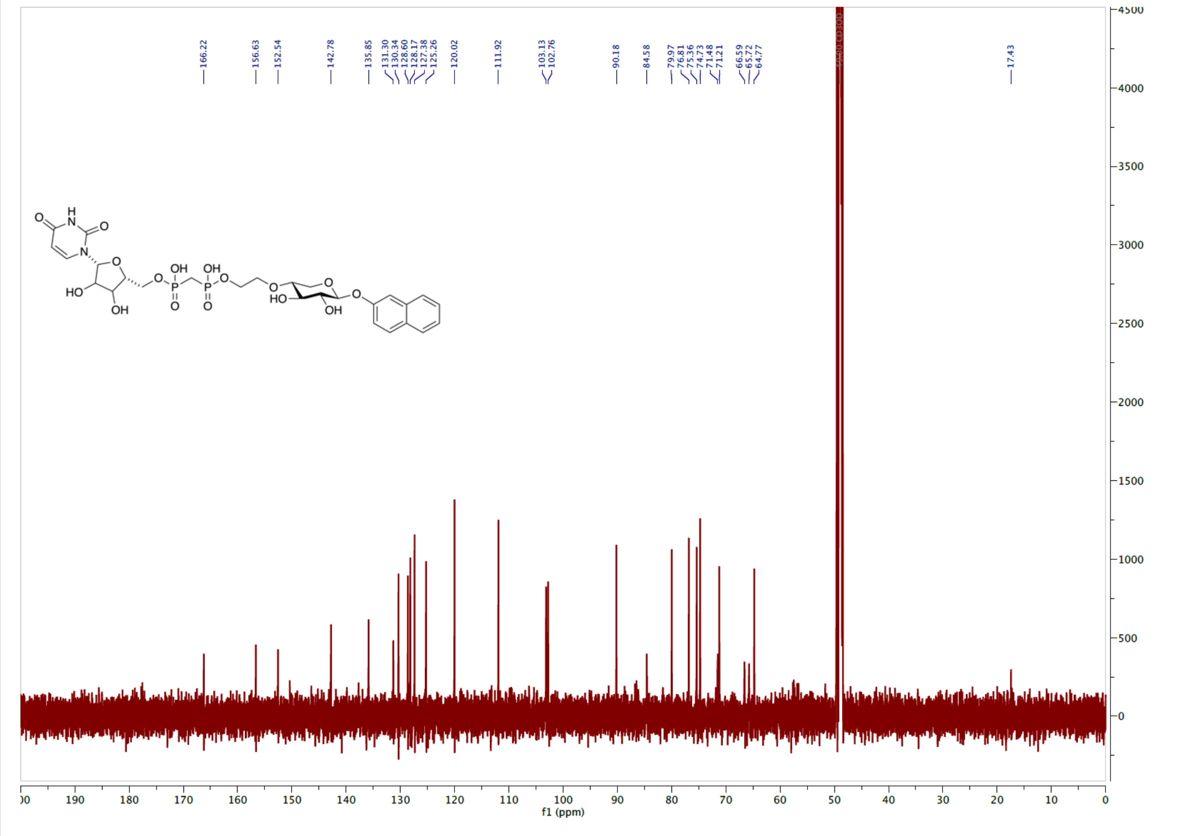

**Figure S5** – $^{13}$C-spectra of compound **1**

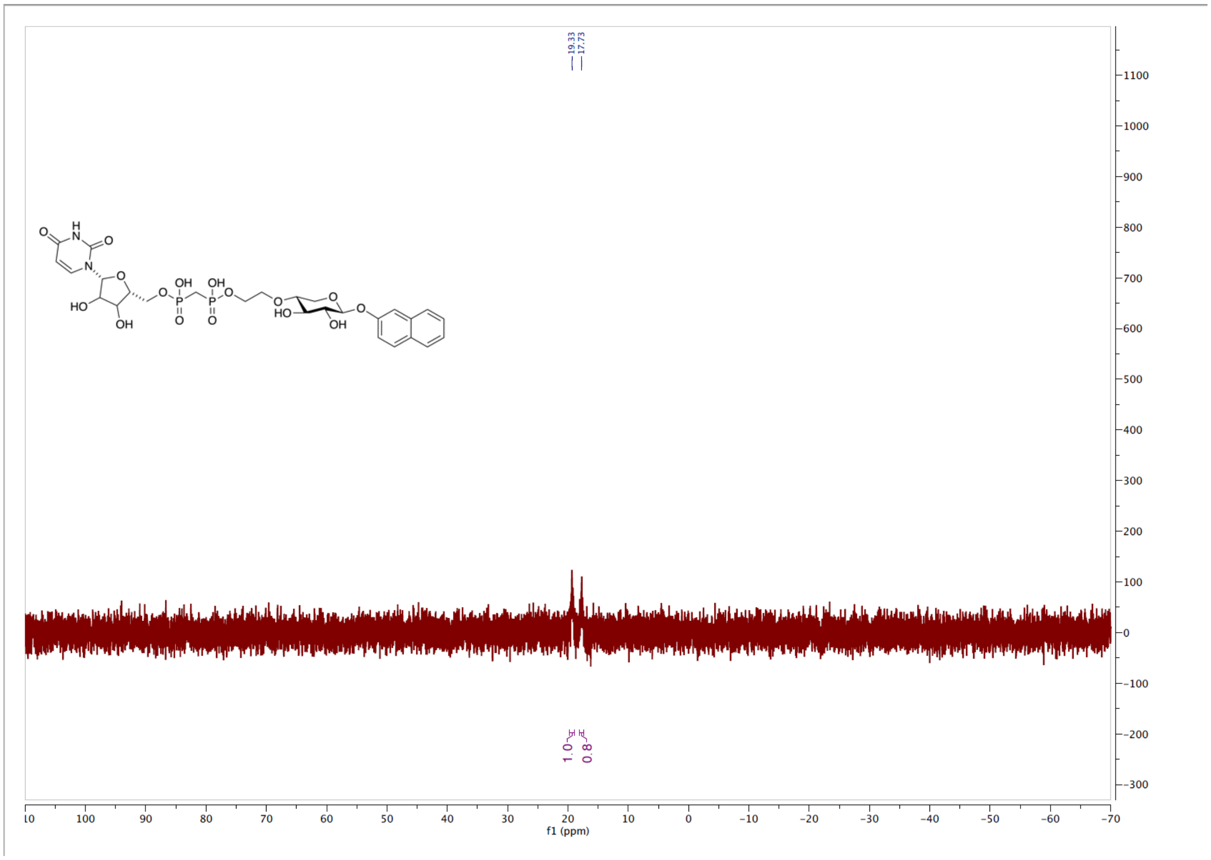

**Figure S6** – $^{31}$P-spectra of compound **1**

## $^1$H-, $^{13}$C-, and $^{31}$P-NMR of compound 2 (Figure S7-S9)

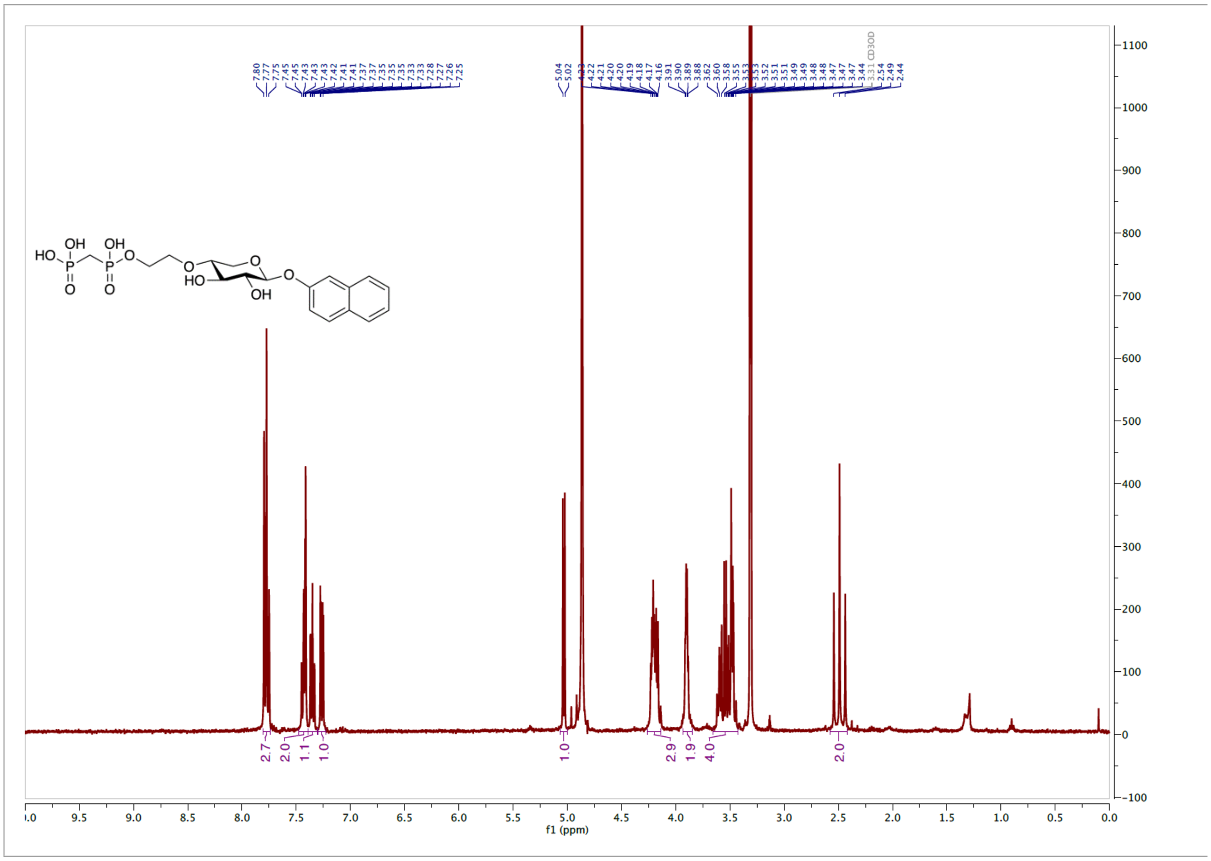

**Figure S7** – $^1$H-spectra of compound **2**

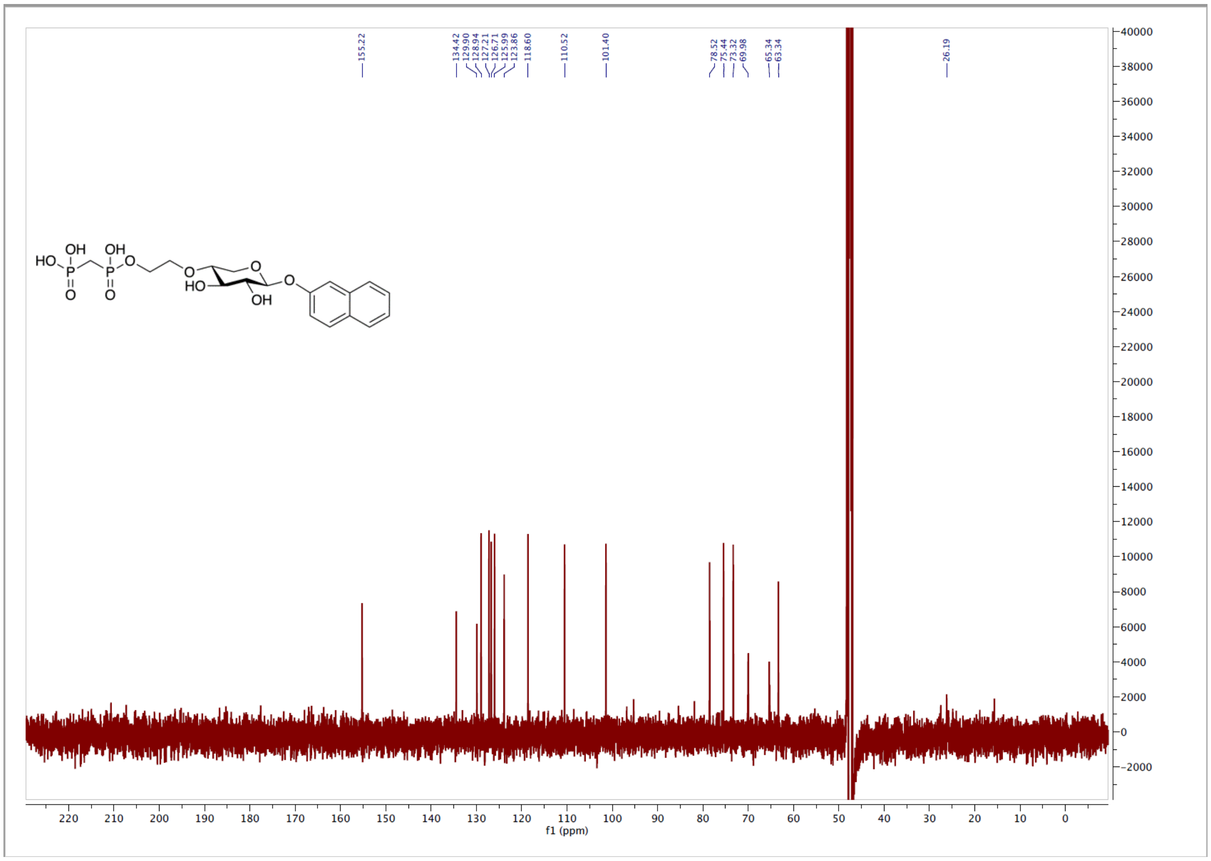

**Figure S8** – $^{13}$C-spectra of compound **2**

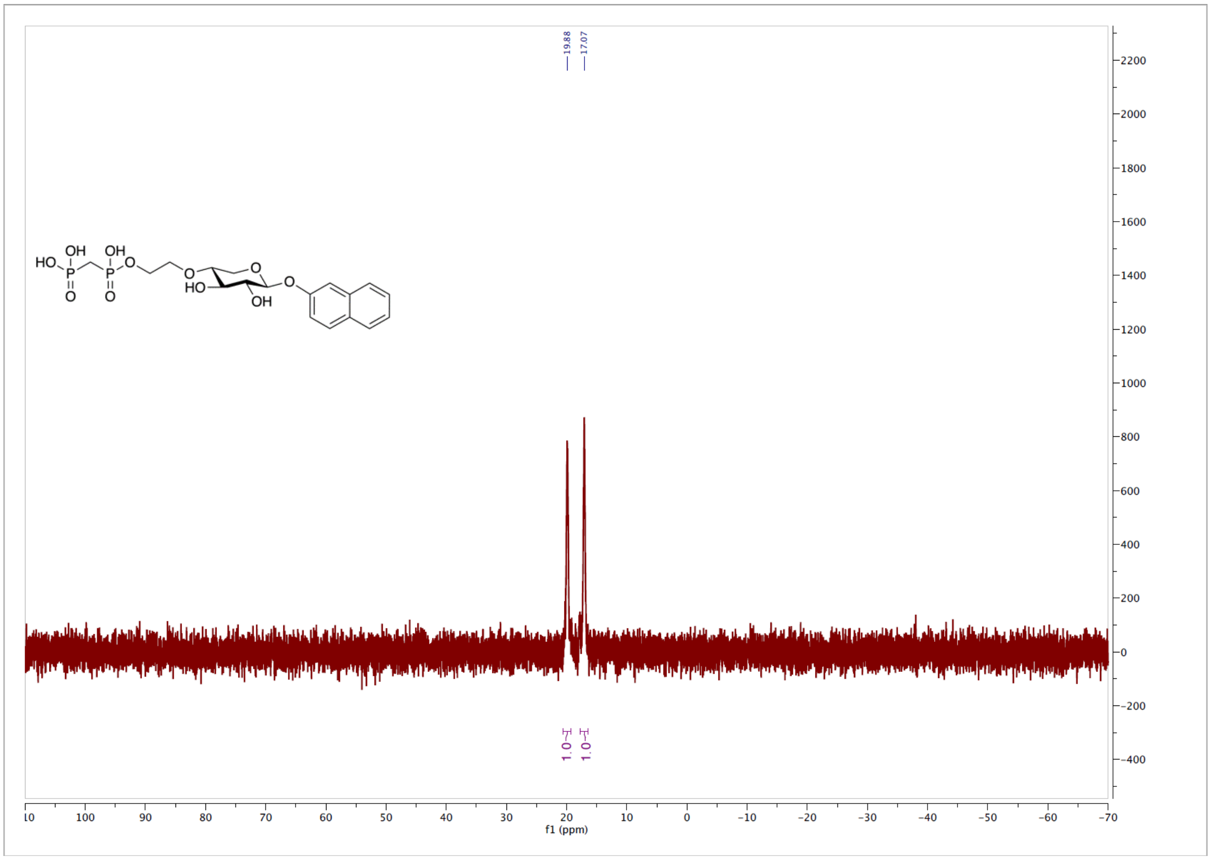


**Figure S9** – $^{31}$P-spectra of compound **2**

## $^1$H- and $^{13}$C-NMR of compound 3 (Figure S10-S11)

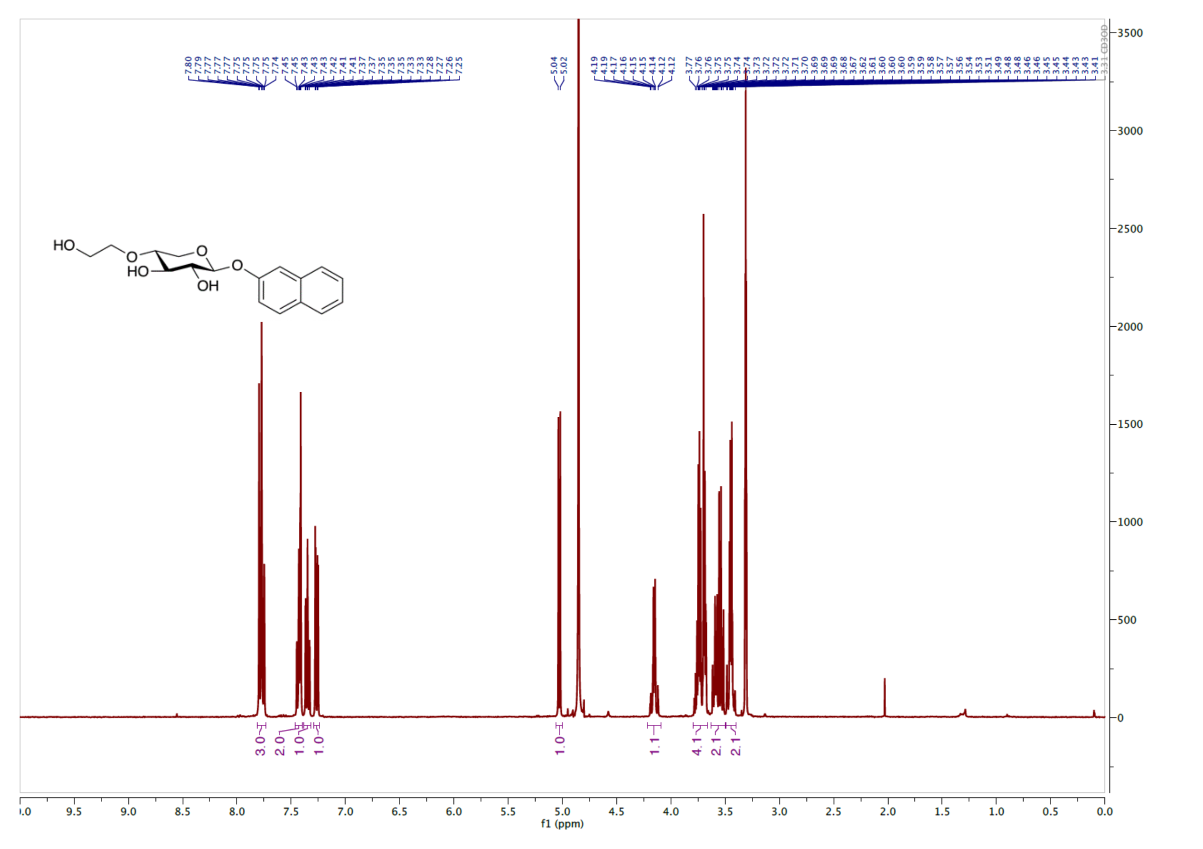


**Figure S10** – $^1$H-spectra of compound **3**

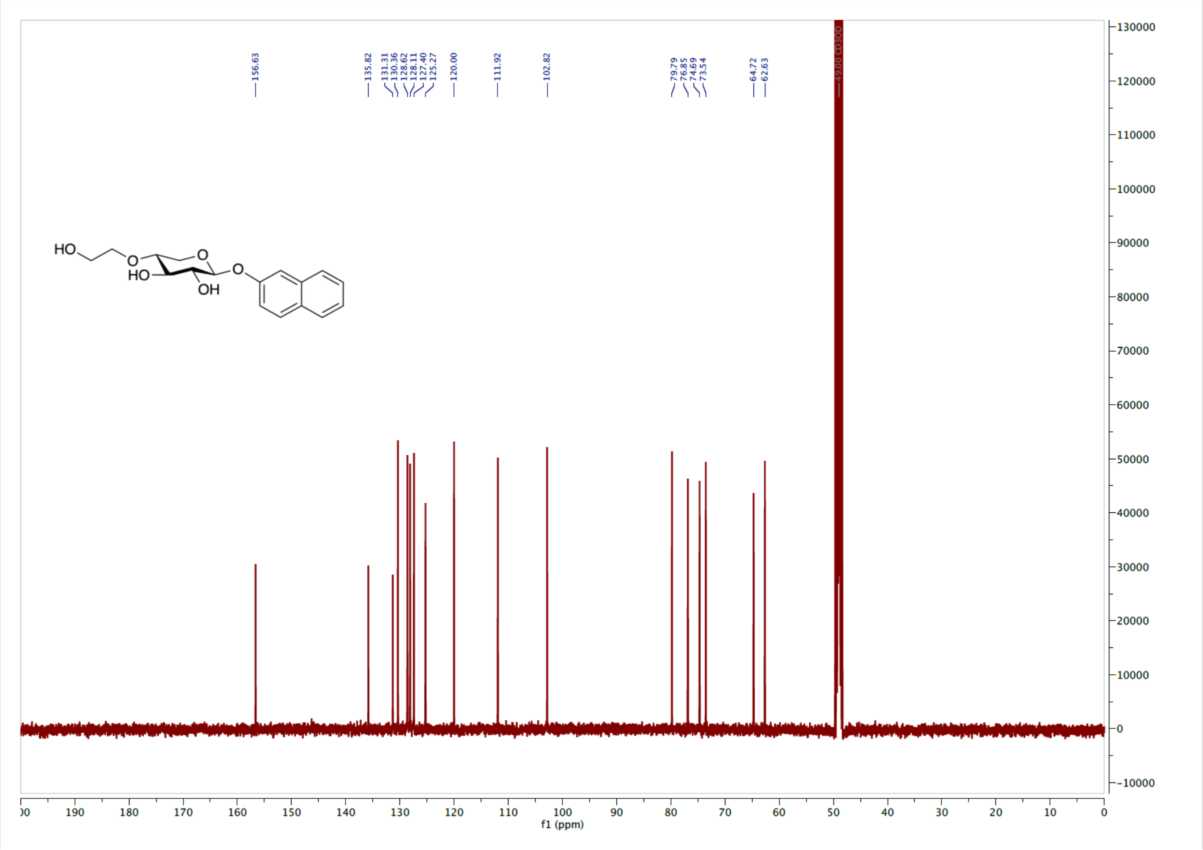


**Figure S11** – $^{13}$C-spectra of compound **3**

## $^{1}$H-, $^{13}$C-, and $^{31}$P-NMR of compound 4 (Figure S12-S14)

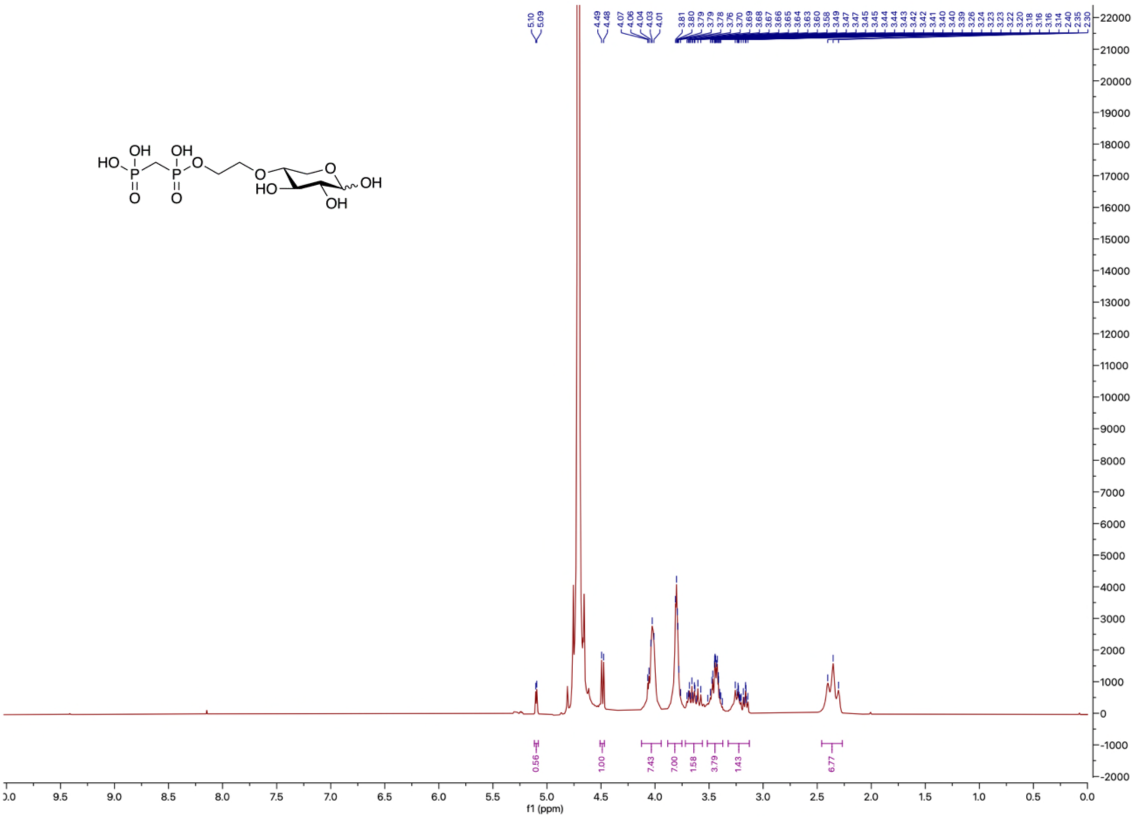


**Figure S12** – $^{1}$H-spectra of compound **4**

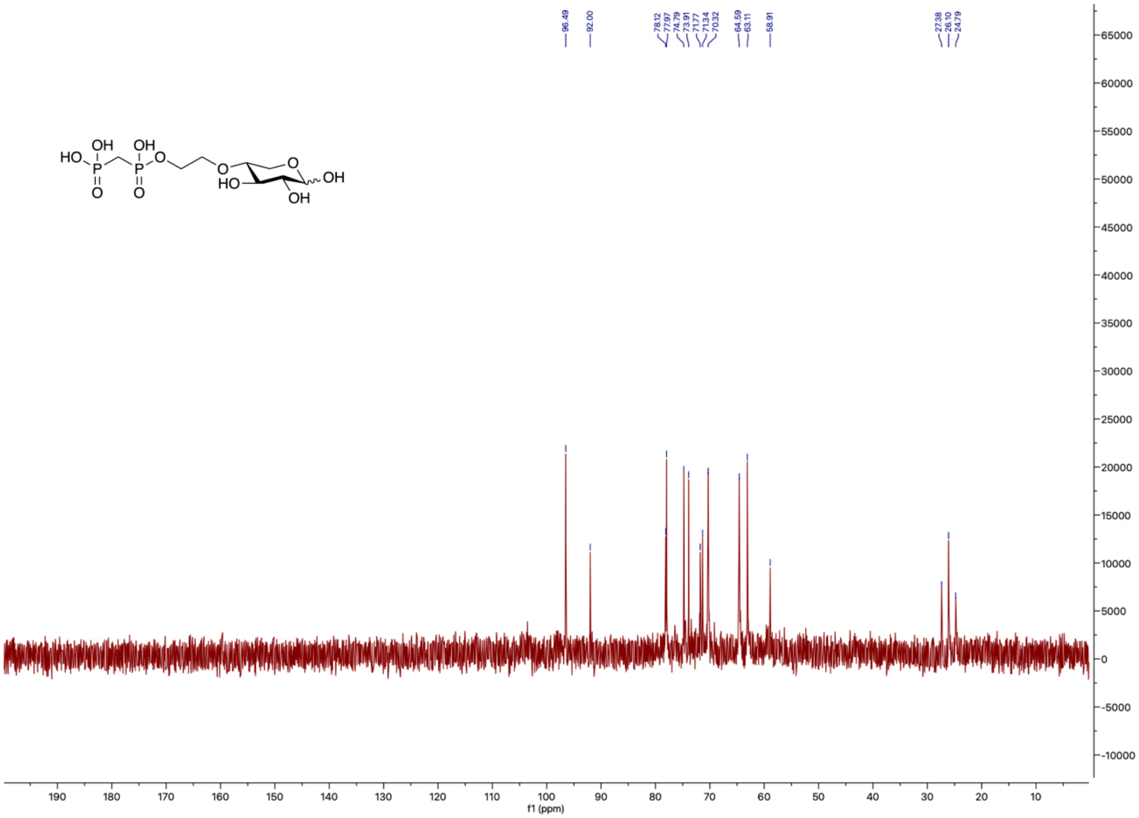


**Figure S13** – $^{13}$C-spectra of compound **4**

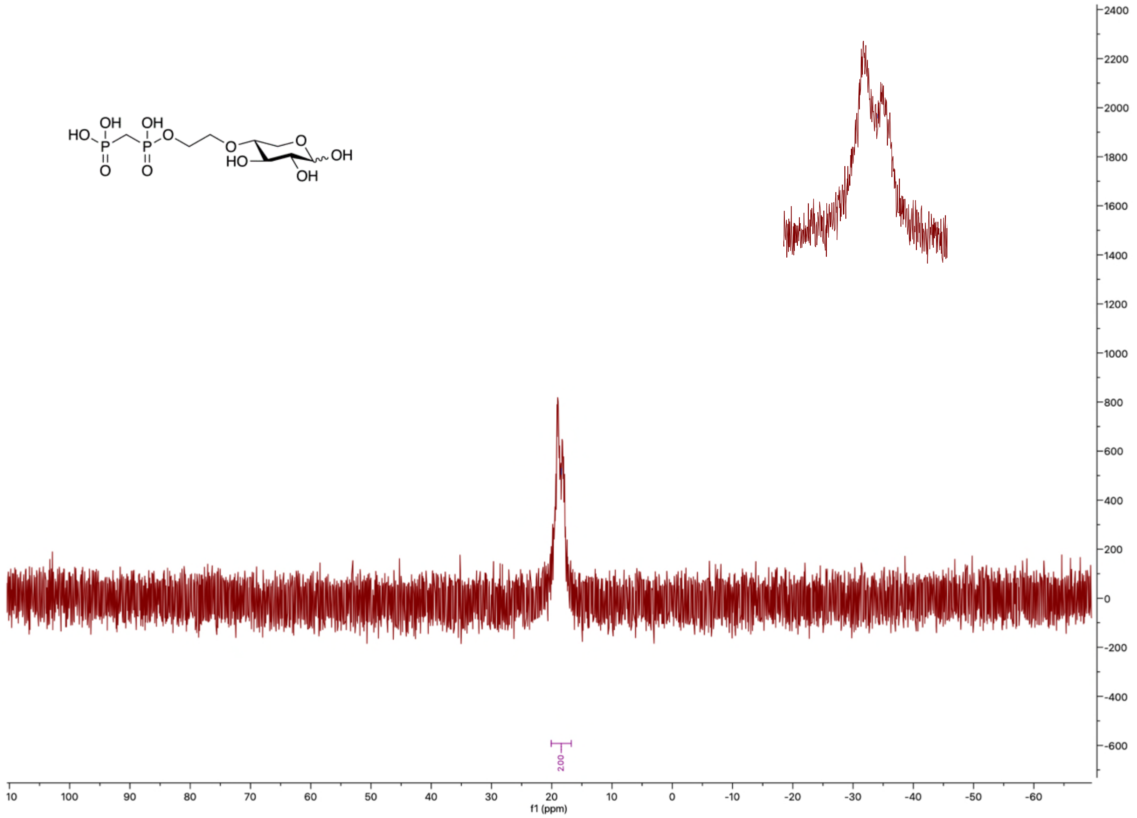

**Figure S14** – $^{31}$P-spectra of compound **4**

## $^{1}$H- and $^{13}$C-NMR of compound 6 (Figure S15-S16)

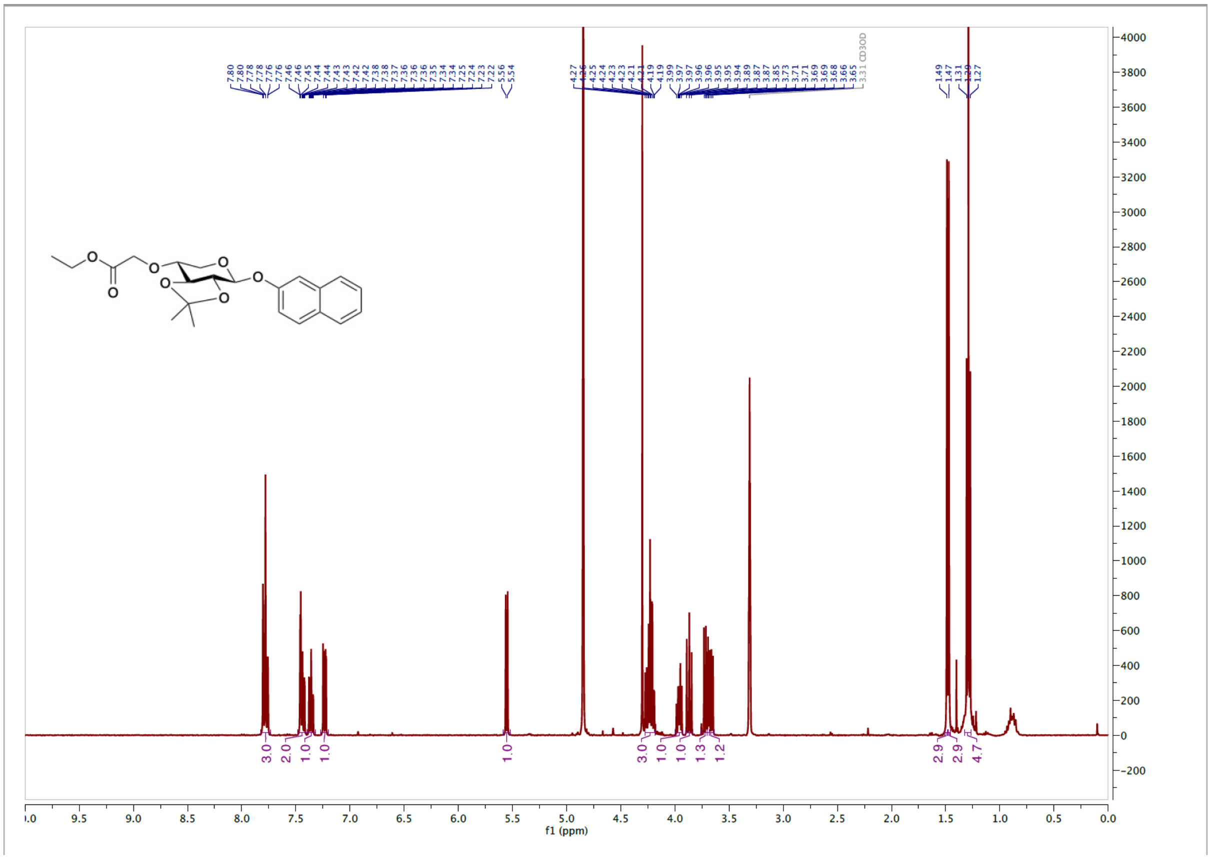

**Figure S15** – $^{1}$H-spectra of compound **6**

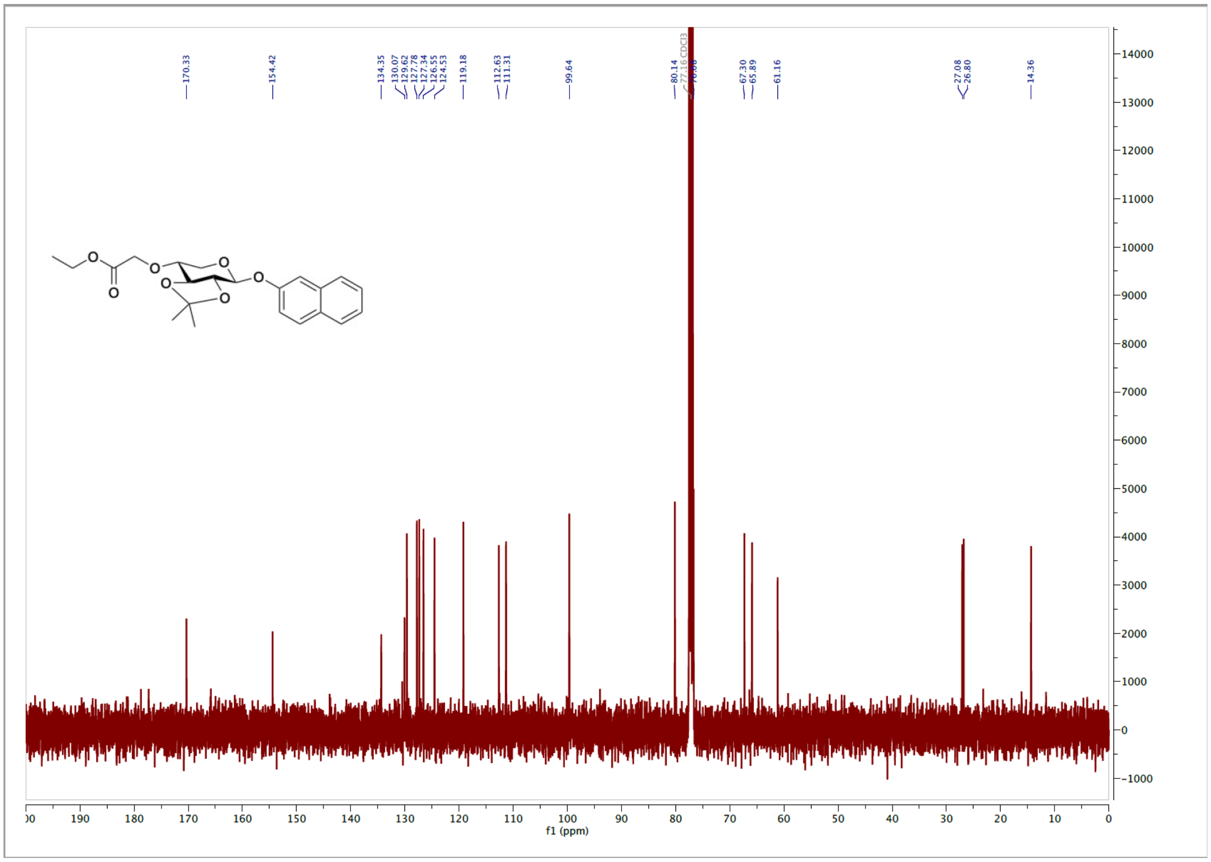

**Figure S16** – $^{13}$C-spectra of compound **6**

## $^{1}$H- and $^{13}$C-NMR of compound 7 (Figure S17-S18)

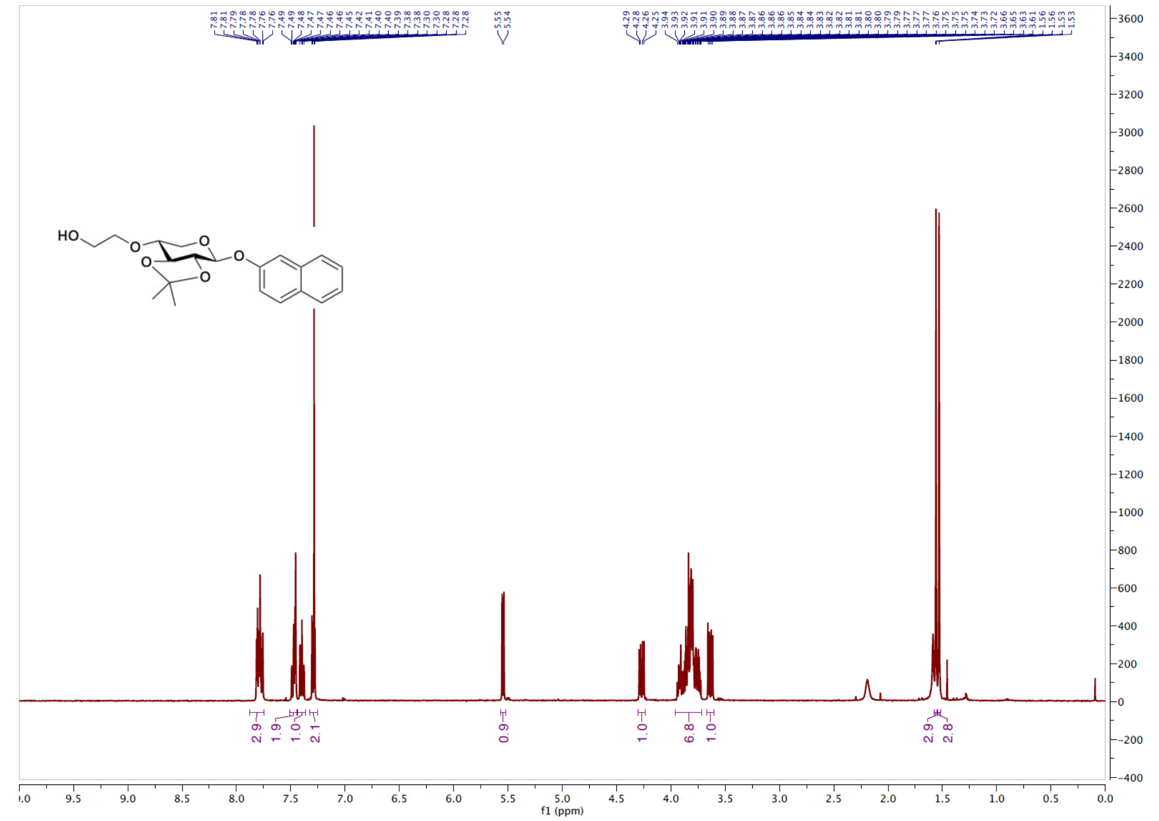

**Figure S17** – $^{1}$H-spectra of compound **7**

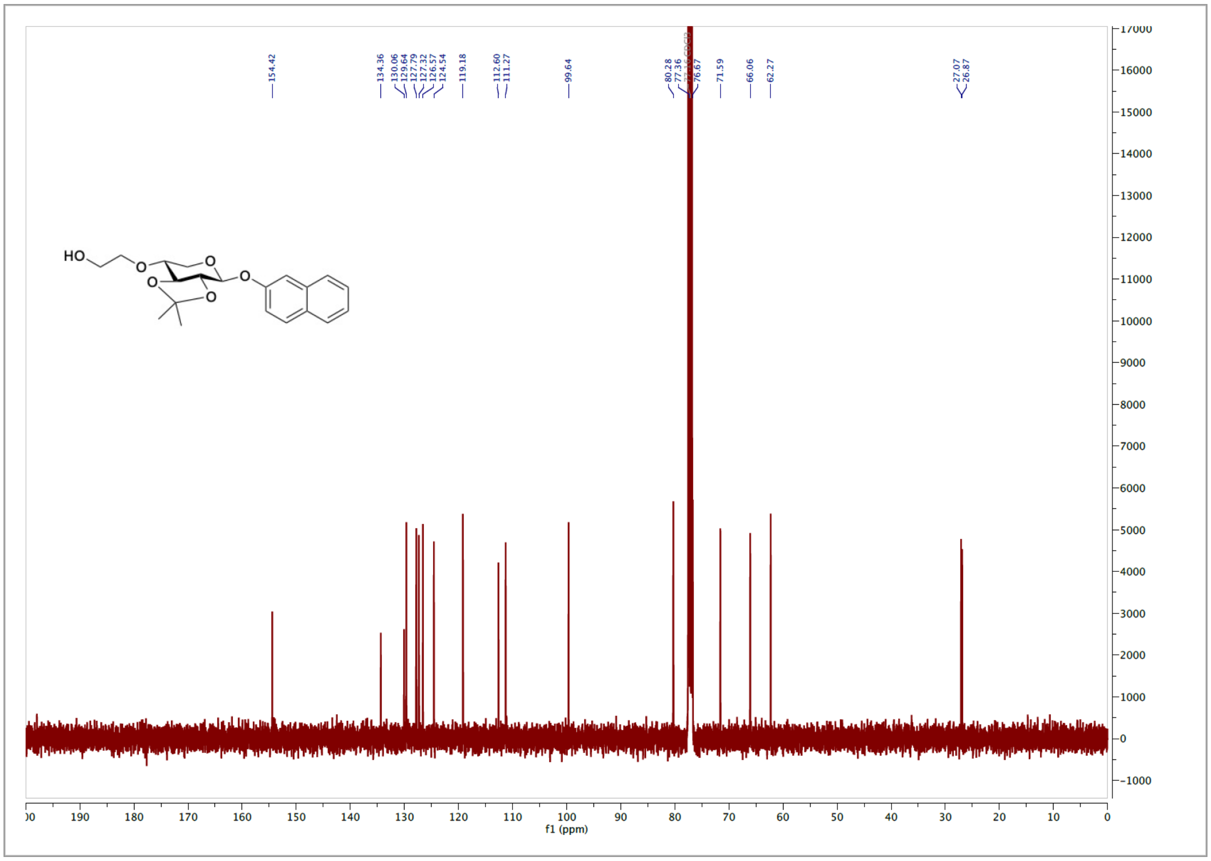

**Figure S18 –** $^{13}$**C-spectra of compound 7**

## STD- and $^1$H-NMR of combinations I – IX of ligands (Figure S19-27)

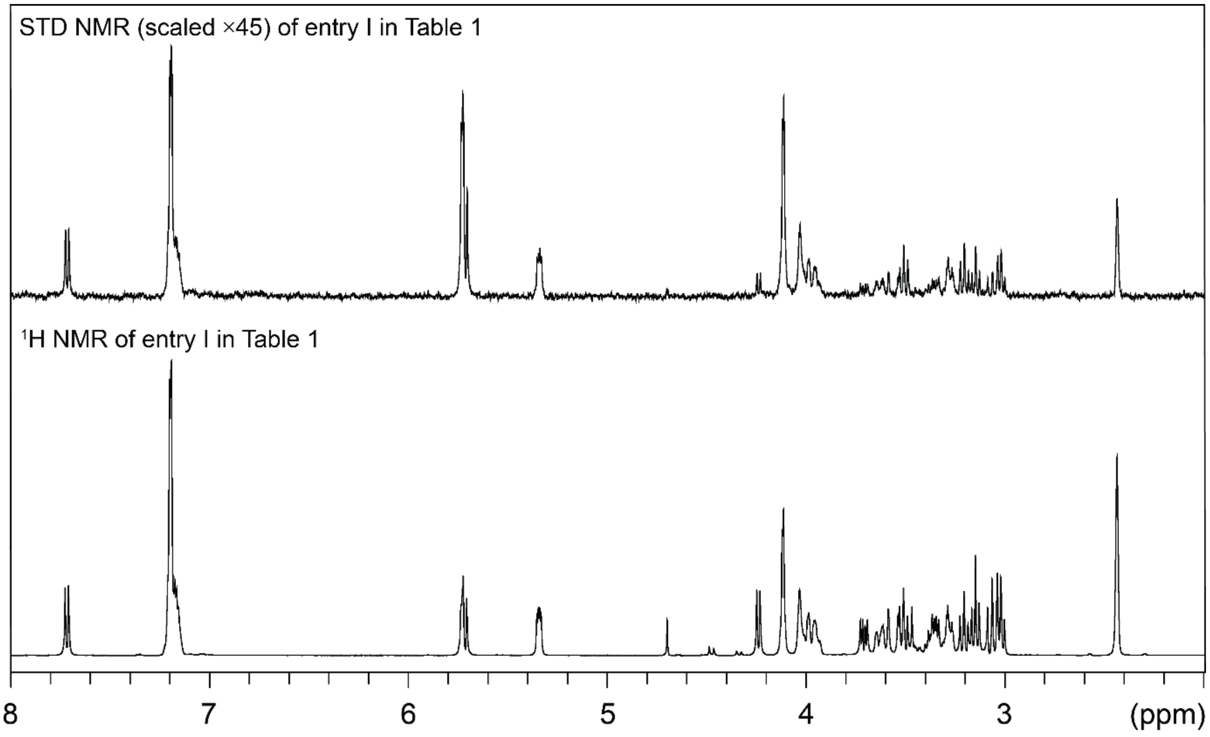


**Figure S19** – STD NMR of entry I and $^1$H-NMR of entry I

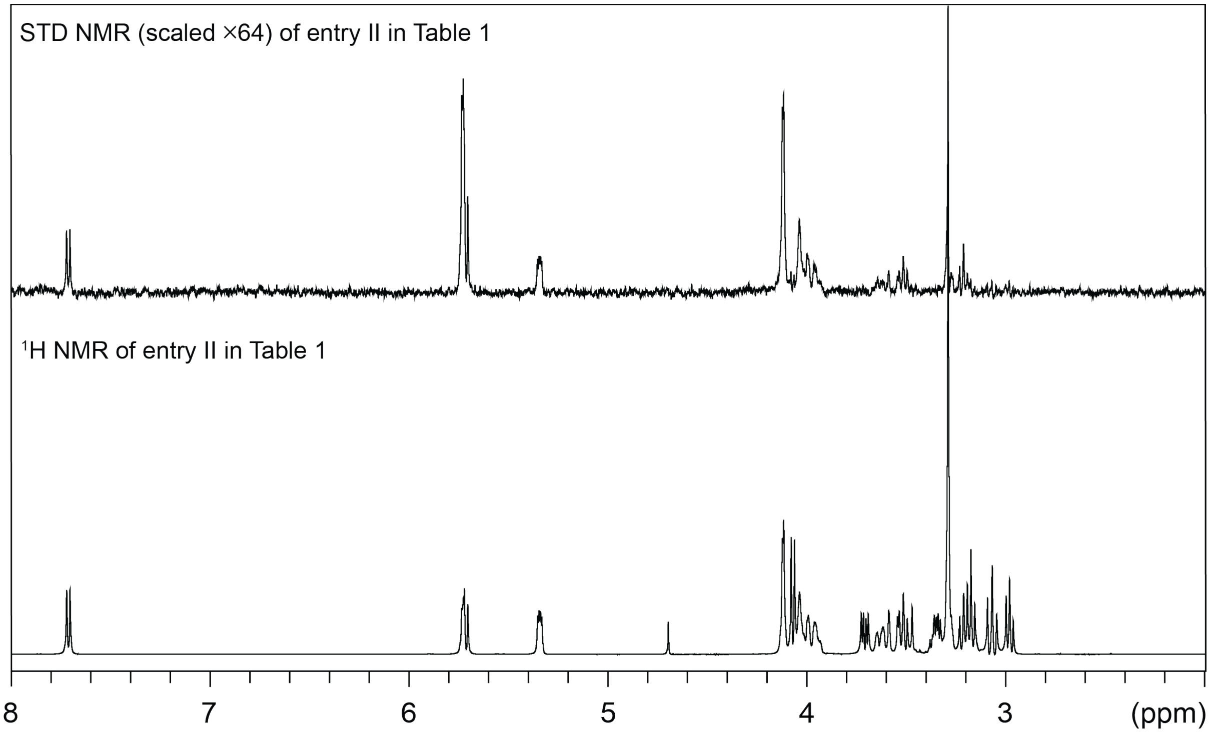


**Figure S20** – STD NMR of entry II and $^1$H-NMR of entry II

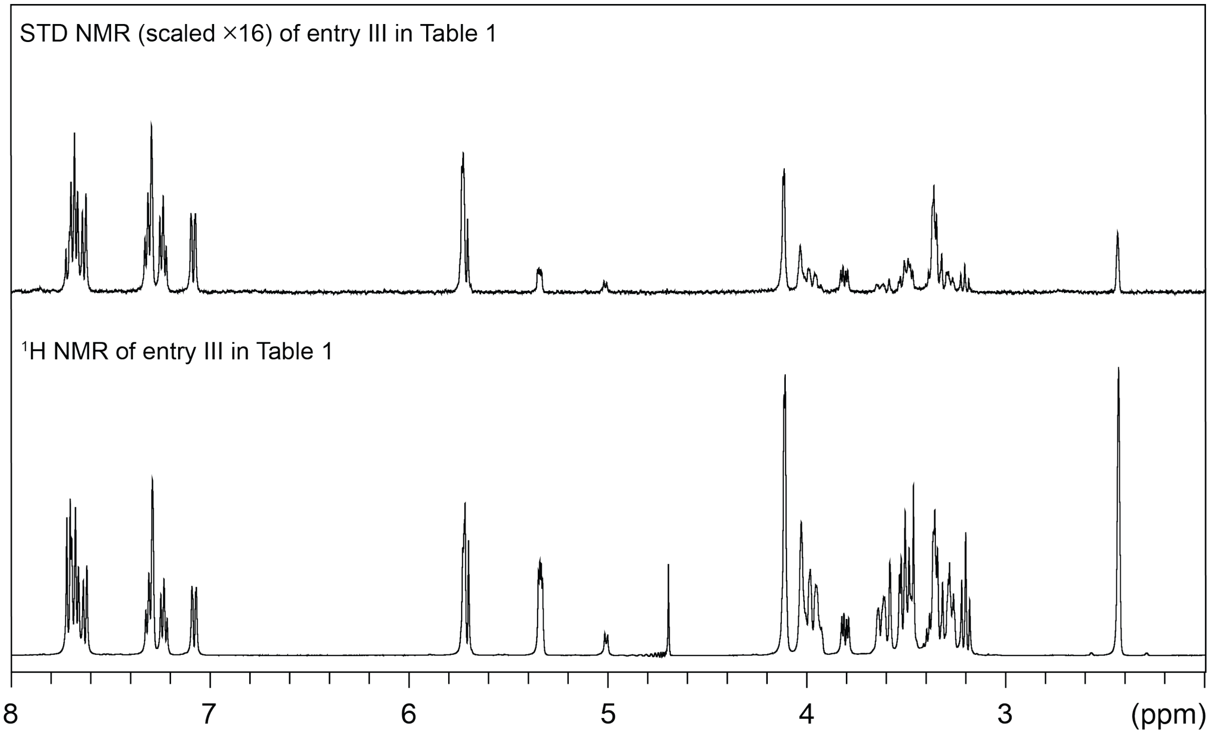


**Figure S21** – STD NMR of entry III and $^1$H-NMR of entry III

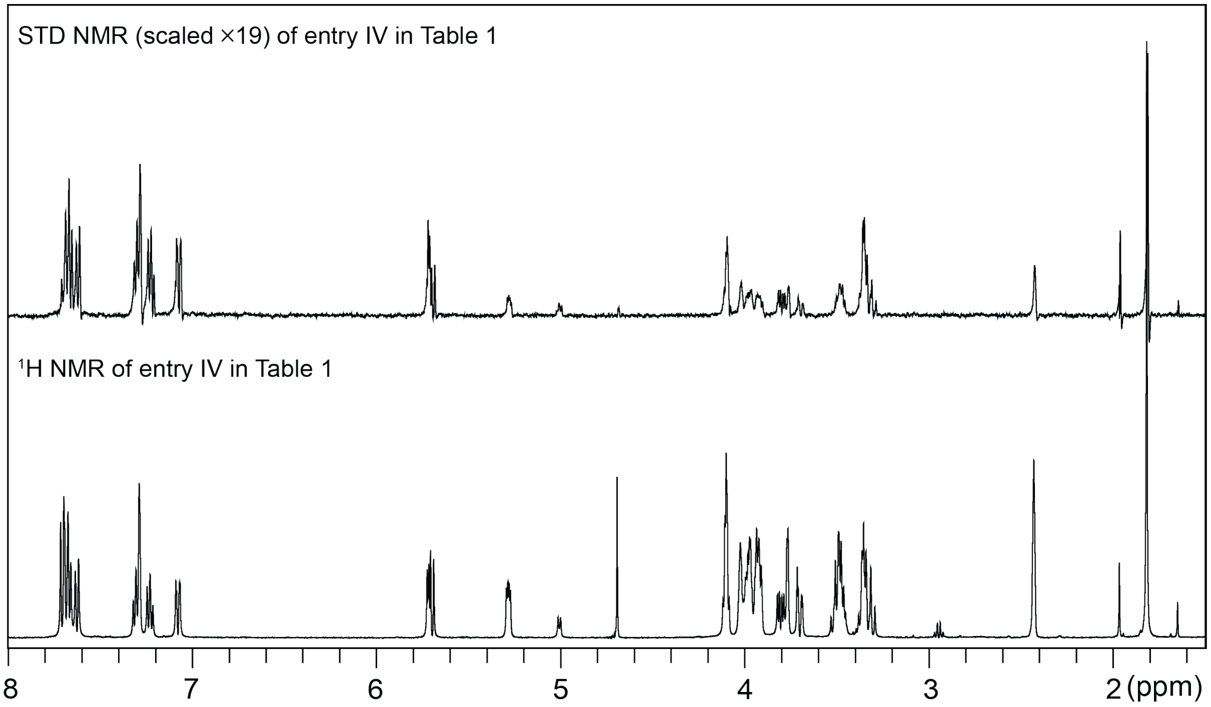


**Figure S22** – STD NMR of entry IV and $^1$H-NMR of entry IV

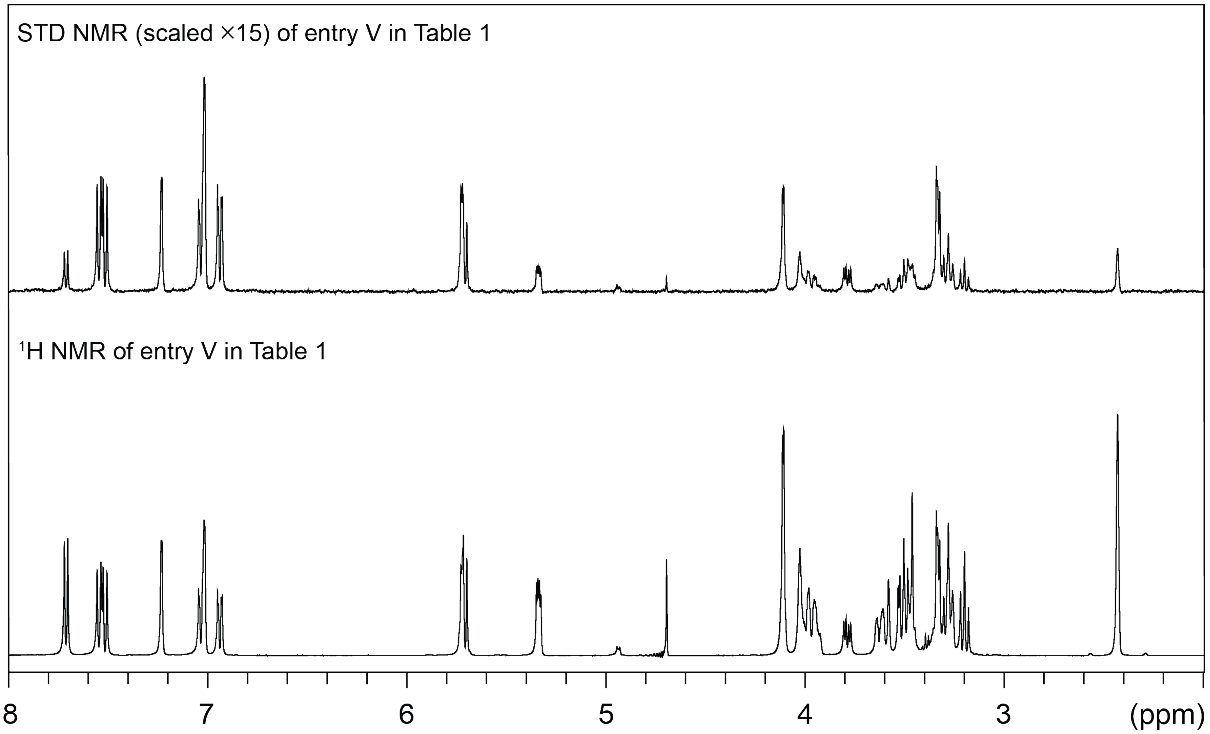


**Figure S23** – STD NMR of entry V and $^1$H-NMR of entry V

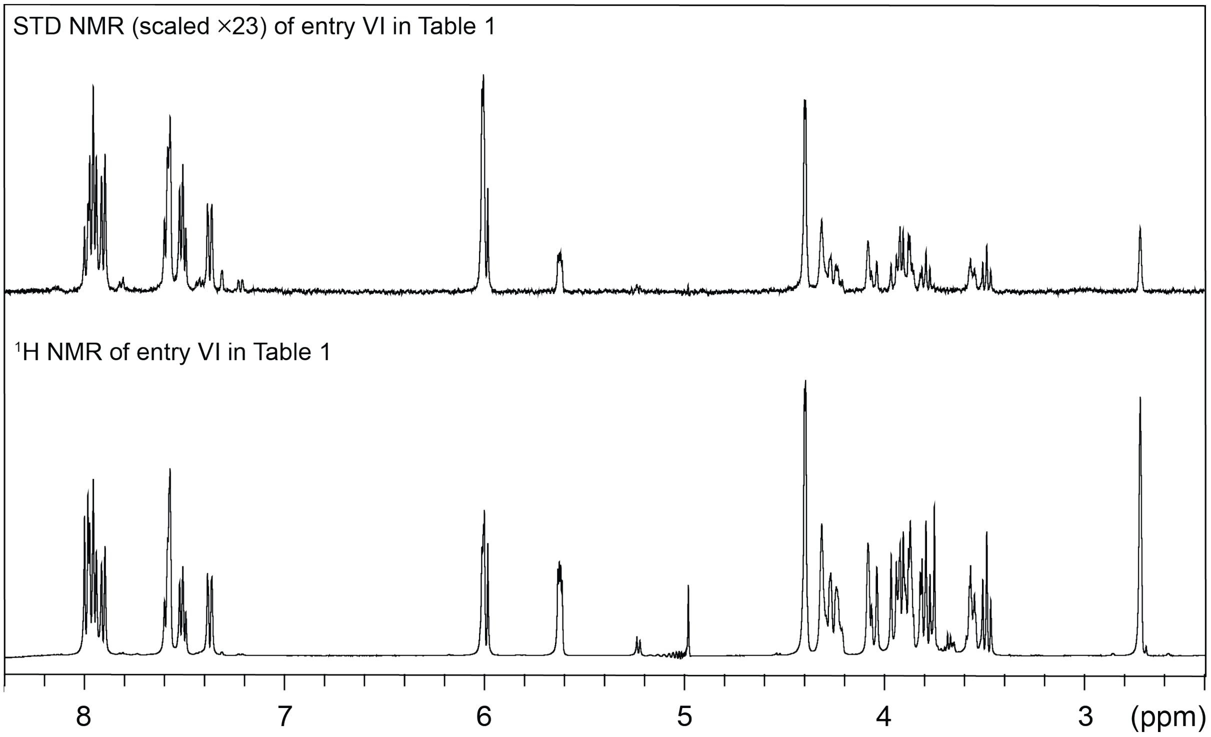


**Figure S24** – STD NMR of entry VI and $^1$H-NMR of entry VI

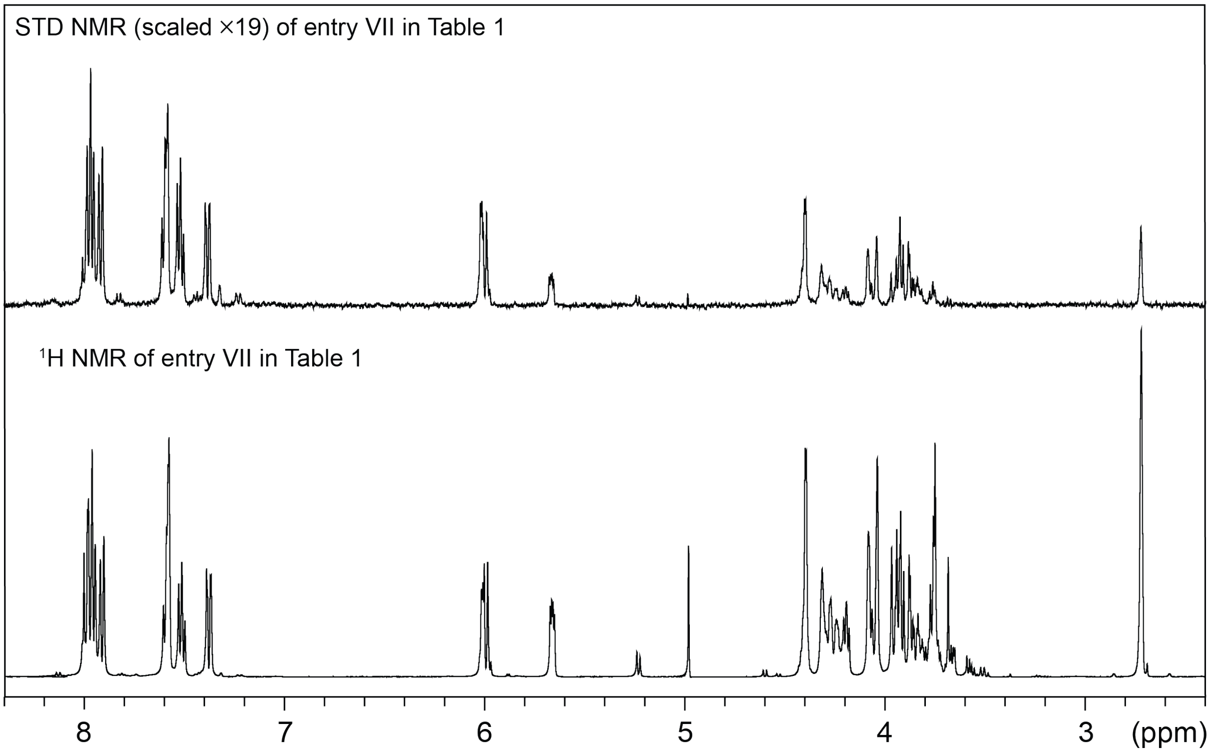


**Figure S25** – STD NMR of entry VII and $^{1}$H-NMR of entry VII

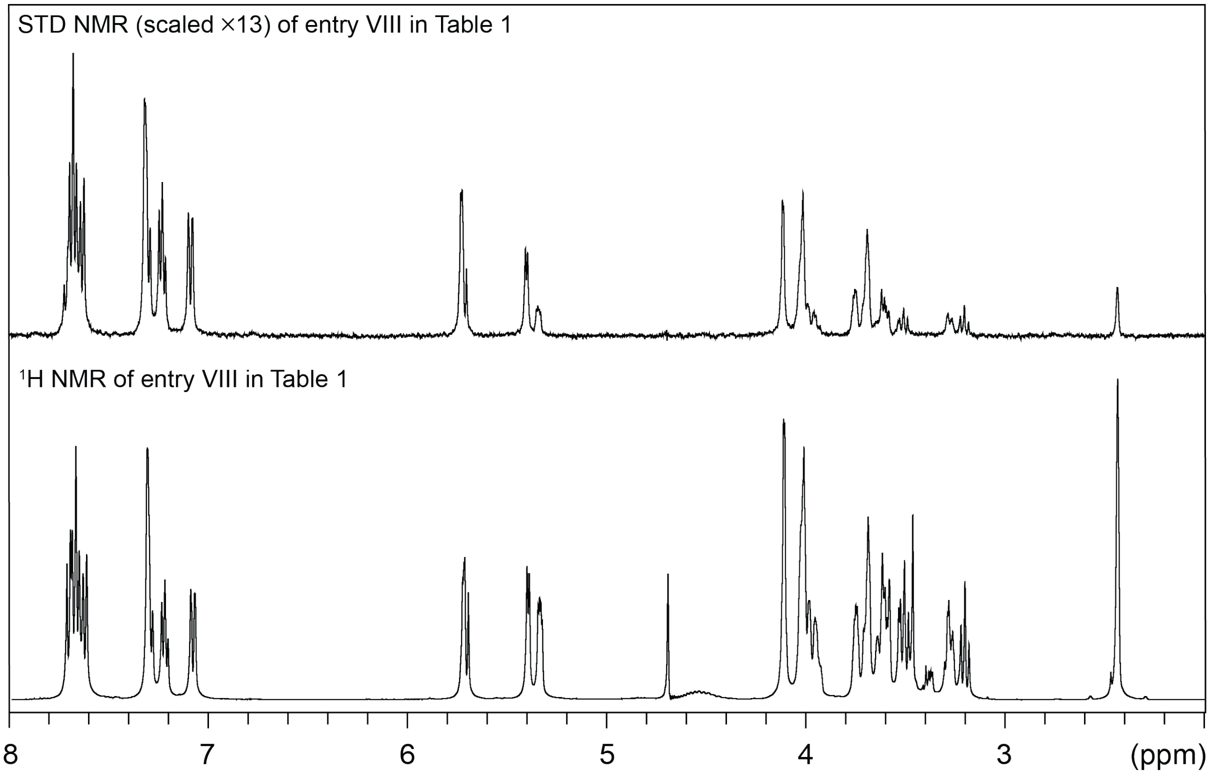


**Figure S26** – STD NMR of entry VIII and $^{1}$H-NMR of entry VIII

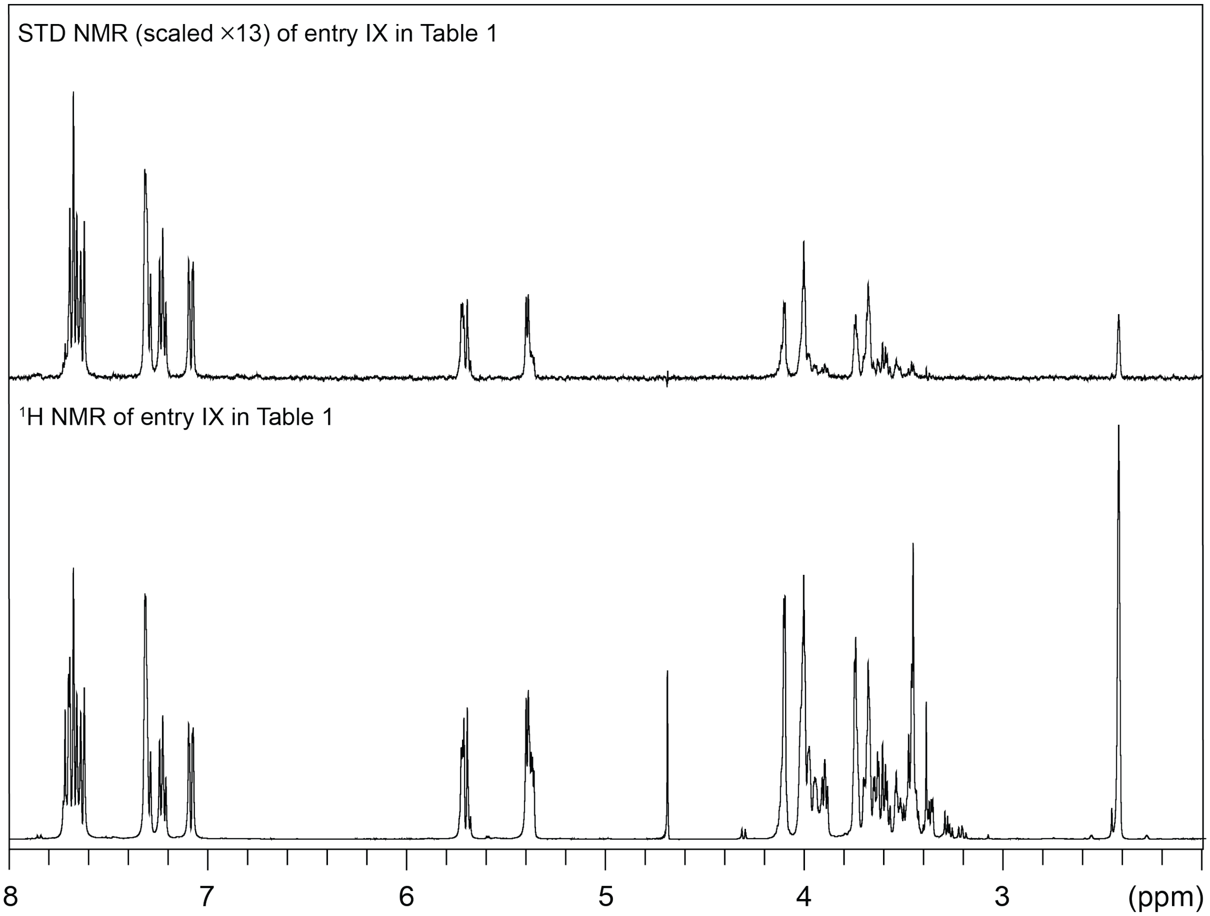


**Figure S27** – STD NMR of entry IX and $^{1}$H-NMR of entry IX
